# Mass Testing With Contact Tracing Compared to Test and Trace for the Effective Suppression of COVID-19 in the United Kingdom: Systematic Review

**DOI:** 10.2196/27254

**Published:** 2021-04-12

**Authors:** Mathew Mbwogge

**Affiliations:** 1 London School of Hygiene & Tropical Medicine London United Kingdom

**Keywords:** COVID-19, SARS-CoV-2, test and trace, universal testing, mass testing, contact tracing, infection surveillance, prevention and control, review

## Abstract

**Background:**

Making testing available to everyone and tracing contacts might be the gold standard to control COVID-19. Many countries including the United Kingdom have relied on the symptom-based test and trace strategy in bringing the COVID-19 pandemic under control. The effectiveness of a test and trace strategy based on symptoms has been questionable and has failed to meet testing and tracing needs. This is further exacerbated by it not being delivered at the point of care, leading to rising cases and deaths. Increases in COVID-19 cases and deaths in the United Kingdom despite performing the highest number of tests in Europe suggest that symptom-based testing and contact tracing might not be effective as a control strategy. An alternative strategy is making testing available to all.

**Objective:**

The primary objective of this review was to compare mass testing and contact tracing with the conventional test and trace method in the suppression of SARS-CoV-2 infections. The secondary objective was to determine the proportion of asymptomatic COVID-19 cases reported during mass testing interventions.

**Methods:**

Literature in English was searched from September through December 2020 in Google Scholar, ScienceDirect, Mendeley, and PubMed. Search terms included “mass testing,” “test and trace,” “contact tracing,” “COVID-19,” “SARS-CoV-2,” “effectiveness,” “asymptomatic,” “symptomatic,” “community screening,” “UK,” and “2020.” Search results were synthesized without meta-analysis using the direction of effect as the standardized metric and vote counting as the synthesis metric. A statistical synthesis was performed using Stata 14.2. Tabular and graphical methods were used to present findings.

**Results:**

The literature search yielded 286 articles from Google Scholar, 20 from ScienceDirect, 14 from Mendeley, 27 from PubMed, and 15 through manual search. A total of 35 articles were included in the review, with a sample size of nearly 1 million participants. We found a 76.9% (10/13, 95% CI 46.2%-95.0%; *P*=.09) majority vote in favor of the intervention under the primary objective. The overall proportion of asymptomatic cases among those who tested positive and in the tested sample populations under the secondary objective was 40.7% (1084/2661, 95% CI 38.9%-42.6%) and 0.0% (1084/9,942,878, 95% CI 0.0%-0.0%), respectively.

**Conclusions:**

There was low-level but promising evidence that mass testing and contact tracing could be more effective in bringing the virus under control and even more effective if combined with social distancing and face coverings. The conventional test and trace method should be superseded by decentralized and regular mass rapid testing and contact tracing, championed by general practitioner surgeries and low-cost community services.

## Introduction

### Background

The United Kingdom’s Test and Trace program has been suboptimal in addressing the testing needs of those infected with SARS-CoV-2 and can hardly be expected to handle its new variant [1]. The panic over rising cases and a potentially more dangerous second wave led to the creation of the National Institute for Health Protection [2]. Other follow-up measures against rising cases have been the implementation of a national lockdown; a tier system; furlough and other support schemes; increased testing; and the approval of the Pfizer, Oxford AstraZeneca, and Moderna vaccines [3,4]. As part of the above, about 56 million tests were performed by January 10, 2021, with about 1.3 million vaccinated [5]. To meet testing needs, the United Kingdom plans to launch the £100-billion “moonshot” program. This program will perform optimally only if tests are delivered based on infections rather than on symptoms in controlling the pandemic [6,7]. According to the Director-General of the World Health Organization, “You cannot fight a fire blindfolded. And we cannot stop this pandemic if we don’t know who is infected” [8]. Knowledge of infections could better inform public policy and facilitate the equitable rollout of vaccines. While we remain hopeful that vaccines will effectively speed up or provide herd immunity, it is important not to lose sight of other control measures like regular, widespread testing. Regular mass testing combined with contact tracing could be a novel control strategy not just to inform vaccination but also to guard against uncertainties arising from any new variant [9].

### Research in Context

Prior to this study, 3 modeling studies implemented in the United Kingdom on mass testing were found. There was also 1 systematic review that evaluated the effectiveness of universal screening for SARS-CoV-2 compared to no screening [10].

This study is the first review, to the best of our knowledge, that sought to evaluate the benefits of mass testing and contact tracing (hybrid strategy) compared to test and trace, to control COVID-19 in the United Kingdom. The reported proportion of asymptomatic cases during mass testing was also explored.

There is an urgent need for a strategy that will identify SARS-CoV-2 carriers when their viral load is high and are most likely to be infectious. Real-time studies are needed to (1) obtain a true picture of disease burden, (2) validate various mass testing options for surveillance, and (3) better inform vaccination programs.

### Conventional Test and Trace

**Figure 1:**
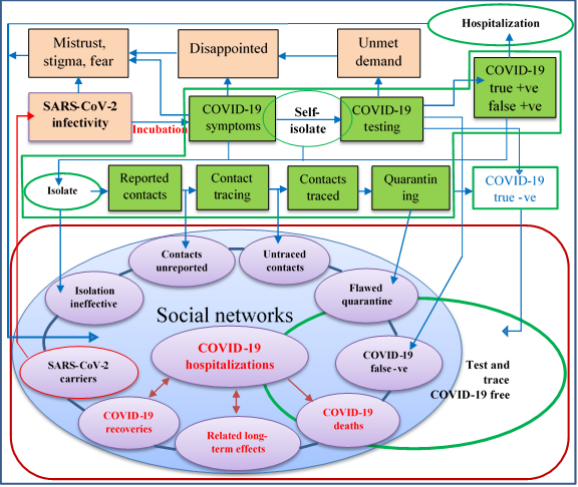
The conventional test and trace system.

Figure 1 shows the traditional test and trace system currently implemented in the United Kingdom, with several possible implications; readers should refer to the UK government website for further details on how the Test and Trace program works [11]. In the face of rising asymptomatic infectivity, the present delivery strategy can be categorized as “the cake not worth the candle,” since the program fails to determine the true burden of the disease.

The following can generally be observed in the conventional system:

1. Individuals who are asymptomatic and presymptomatic are missed [12,13];
2. People are generally afraid of quarantine and may shy away from testing [14];
3. Decisions related to public safety (eg, getting tested) have been shifted to the public;
4. Operational false-positive estimates in the United Kingdom are currently unknown [15];
5. The proportion of daily asymptomatic cases is still not part of the reported national statistics and the true disease burden remains unknown [16];
6. Test and trace depend on self-reported contacts, which may be flawed;
7. Members of the public are hesitant due to data ethics–associated stigma [17];
8. The test and trace strategy is a shift away from universal health coverage in the midst of a pandemic [18];
9. Long travel and other factors are barriers to accessing sample collection centers;
10. There seems to be an apparent mix-up between “sample collection centers” and “testing centers.”

### The “Infectivity Problem” of COVID-19

The “infectivity problem” can be summarized into (1) the test ramp-up controversy, (2) test and trace system leakages, (3) the time-to-test paradox, (4) inequitable test delivery, and (5) test and trace system delays.

#### Test Ramp-up Controversy

This refers to the heated discussion and lockdown-related antagonism expressed by the public regarding the undesired positive correlation, which was presumed inverse, between testing capacity and COVID-19 cases. The supposed endgame of test ramp-up was to contain the virus, but countries have found themselves in the opposite situation. This may be due to more cases now being detected as a result of increased testing or because testing is not comprehensive and early enough to outweigh viral shedding. This may culminate into the United Kingdom’s “operation moonshot” controversy if the testing rate continues to be less than the infectivity rate [19].

#### Test and Trace System Leakages

Leakage refers to infectious individuals who are not detected. This includes those with either unreported symptoms or not presenting for testing despite being able to, those sent home due to an unavailability of tests, testing conducted on samples of compromised quality, unreported and untraced contacts, false negatives, and noncompliance to isolation and quarantine rules [20-22].

#### Time-to-Test Paradox

This refers to the conflicting interest of whether to test before symptom onset or upon reported symptoms. The Test and Trace program has been designed not to test people at the very early stages of infection for fear of missing out on the very cases it is meant to detect. The same is true when people are tested late [23,24]. A hidden “giant” within this paradox and a major contributor to transmissions is asymptomatic and presymptomatic infectivity. Research suggests that the serial interval of COVID-19 is shorter than the incubation period, indicating a possible infectivity multiplier effect before the onset of symptoms [25,26]. This is further compounded by the currently unknown operational false negatives [15].

#### Inequitable Test Delivery

This refers to testing that is not only being selective but is also not being delivered at the point of care. As a result, a major group of the public is eliminated. This has led to the lack of a comprehensive understanding of disease behavior.

#### Test and Trace System Delays

The problem includes delays in testing those reporting symptoms, test-to-results delays, and time lapses in contact tracing. These system delays have led to increasing infections in the face of delivering the highest number of tests in Europe [27]. A disease that is as deadly as the present one does not tolerate turnaround time and mitigation program mistakes, the biggest of which has been the neglect of asymptomatic infectivity.

## Methods

### Study Objectives

In this study, we compared the strategy of mass testing and contact tracing with the conventional test and trace method in the control of COVID-19 in the United Kingdom. Mass testing and contact tracing is one proactive way of testing individuals irrespective of symptoms to detect infections, track their contacts, and break the transmission circuit of SARS-CoV-2 in a timely manner [28,29].

This study’s objective was twofold. We aimed (1) to evaluate the evidence of mass test and trace compared to conventional test and trace in the suppression of community transmissions of COVID-19 and (2) to find out the proportion of asymptomatic carriers during mass testing interventions.

The primary and secondary research questions are (1) is there evidence that testing irrespective of symptoms combined with tracing could suppress SARS-CoV-2 infections better than symptom-based testing and tracing? and (2) what is the proportion of asymptomatic carriers of SARS-CoV-2 reported during mass testing interventions?

### Database Search

#### Search Strategy

A literature search was performed on September 9, 2020, and constantly refreshed through December 22, 2020. The search involved all articles in English published in 2020, including gray literature. Search terms in Google Scholar included “[UK] [effectiveness of mass testing] [COVID-19] [SARS-CoV-2] [contact OR tracing] [contact tracing] [effectiveness of test and trace] –Animals –Influenza –HIV –Cancer.” The search was restricted to the year 2020.

An advanced search was performed in ScienceDirect for “[test and trace] OR [contact tracing] AND [COVID-19] AND [SARS-CoV-2] AND [asymptomatic] AND [symptomatic] OR [screening for SARS-CoV-2] OR [mass testing for SARS-CoV-2]” with article titles terms “[UK] AND [test and trace] OR [contact tracing] OR [community screening for SARS-CoV-2] OR [mass testing for SARS-CoV-2].” The search was restricted to the year 2020.

A search in PubMed included “((((((((mass testing for COVID-19 and “contact tracing”) OR (mass testing for SARS-CoV-2 and “contact tracing”)) OR (“test and trace”)) OR (“mass testing” and “symptom-based testing”)) NOT (Animals)) NOT (HIV)) NOT (Influenza)) NOT (Ebola)) NOT (Cancer).”

Finally, a search for “mass testing for COVID-19” AND “contact tracing for COVID-19” OR “mass testing for SARS-CoV-2” AND “contact tracing for SARS-CoV-2” was performed in Mendeley.

#### Eligibility Criteria and Exclusion

##### Eligibility

The population of interest included persons infected with SARS-CoV-2 who were either symptomatic or asymptomatic. The intervention of interest was mass testing irrespective of symptoms and tracing contacts. The comparison was a test and trace strategy based on symptoms. We were interested in studies evaluating effectiveness, cost-effectiveness, safety, acceptability, and equity under the primary research question, and the proportion of asymptomatic cases under the secondary research question. Studies that did not include contact tracing but compared testing irrespective of symptoms and symptom-based testing were also included under the primary research question.

##### Exclusion

Articles were excluded if they were published before the year 2020, were not in English, had inaccessible full texts, were not related to COVID-19, focused on nonhuman subjects, and were not related to mass testing. Given that this review was about detecting people currently infected, we excluded antibody studies. We also excluded editorials, theses, protocols, and news articles.

#### Selection and Publication Bias

The preferential publication of studies was counteracted by ensuring that our search included gray literature. Missing data effect verification was performed by searching for gray literature that sought to compare the effectiveness of the intervention to the control [30].

### Data Management

#### Data Extraction

We performed a detailed screening of the extracted data for individual studies. Extracted data included the study date, author, setting, study design, study objective, type of intervention, outcome, type of participants, strategies used, assumptions, data analysis, results, study limitations, and bias.

#### Criteria for Grouping Studies

Following our study objective, studies for synthesis were grouped according to study outcomes. This was done to help capture the studies whose interventions were geared toward evaluating effects on outcomes of interest [31]. This also facilitated the synthesis of results according to the research questions.

#### Data Quality Assessment

Review findings were synthesized thematically. The quality of studies was critically appraised using the most recent tools based on study design, following the Public Health Ontario MetQAT (Meta Quality Appraisal Tool) 1.0 [32,33]. The methodology and risk of bias of modeling studies were assessed using the Relevance and Credibility Assessment of Modeling Studies tool proposed by Caro and colleagues [34]. Cohort studies were assessed using the Critical Appraisal Skills Program (CASP) tool [35]. The Specialist Unit for Review Evidence (SURE) tool was used to assess cross-sectional studies [36]. Studies were grouped into 6 main categories according to study outcomes, as outlined in the eligibility criteria, for easy analysis and synthesis. The quality of evidence generated by different studies was assessed using the Grading of Recommendations Assessment, Development and Evaluation (GRADE) tool [37].

#### Standardized and Synthesis Metrics

The direction of effect was used as the standardized metric because there was a lack of precision, which was specific to the effect of the intervention and control in the results presented by different studies. This did not permit the calculation of summary statistics [38]. In light of the above, vote counting was the best match in synthesizing the results. A sign test was used to indicate whether there was evidence of an effect or not. Equivocal effects between the intervention and control were considered to be distributed around the null hypothesis of no effect. This study made use of Synthesis Without Meta-Analysis (SWiM) reporting guidelines to report review results [39].

#### Data Presentation and Visualization

Tabular and graphical methods were deployed in presenting the results of this study. For the primary objective, the GRADE summary of findings table was used to present the certainty of evidence and a bar chart to present the effect direction of studies. For the secondary objective, forest plots were used to present the proportion of asymptomatic cases of SARS-CoV-2, using an Excel model proposed by Neyeloff et al [40].

#### Criteria for Prioritizing Results

Concerning the primary question, the results of studies that evaluated the effectiveness of the intervention and control within the United Kingdom, with low risk of bias, were prioritized since this was in line with the review objective. Real-time studies were also prioritized as these are more likely to resemble reality.

#### Heterogeneity Assessment

The heterogeneity of studies was assessed following the GRADE risk assessment factors [41]. The lack of a pooled effect size for modeling studies did not warrant us to perform a test for methodological diversity for the primary objective [42]. Regarding the secondary objective, however, variability was assessed by directly observing the confidence intervals on the plotted graphs.

### Active Runs of the Intervention

**Figure 2:**
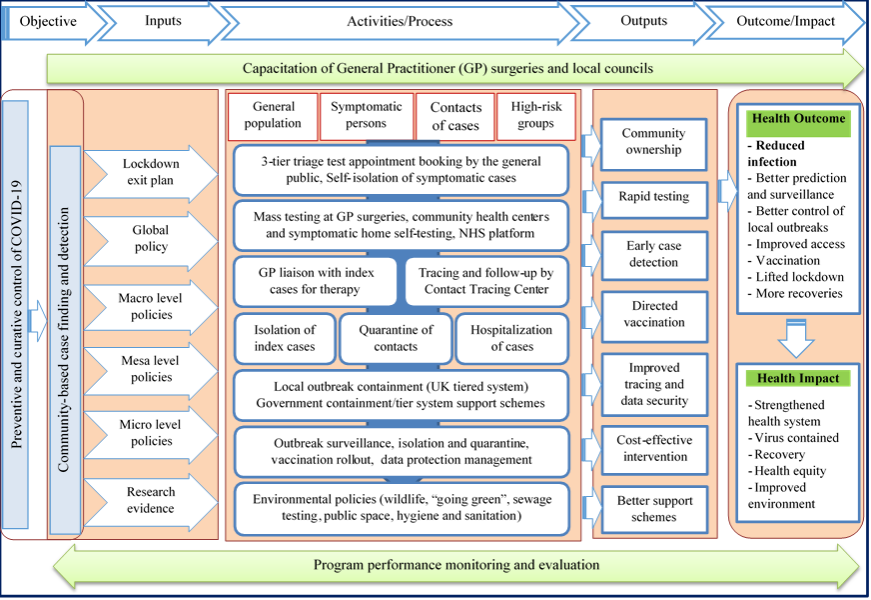
Framework for decentralized mass testing and contact tracing. NHS: National Health Service.

The novel mass test and contact trace strategy (1) extends the present test and trace system to the general public and (2) moves it from laboratory-based to point-of-care settings, thereby enhancing acceptability, accessibility, and equity. A framework is used to explain how the novel strategy could be implemented. This framework is a modification of the one proposed by Lassi et al [43]. Community ownership in the implementation of this strategy requires each individual to be registered with a general practitioner (GP) surgery and the capacitation of GP surgeries to perform routine, open-invitation testing irrespective of symptoms. The strategy equally necessitates the availability of rapid easy-to-run, cost-effective tests and a succinct phasic exit strategy. Strategy inputs include macro policies (fiscal, support schemes, personal protective equipment, hygiene and sanitation, environmental, a tier system, vaccination development and approval, etc), mesa policies (GP capacitation, social gathering, at-risk group, vaccination, etc), and micro policies (testing, health status, personal hygiene, compliance to national guidelines, tracing app acceptability, etc). Routine health checks with GPs have hardly raised concerns around privacy due to trust. Patients find it more reliable and assuring if GPs run testing programs, offer direct vaccination and therapy to those that have tested positive, and request those with positive test results to report their contacts on the National Health Service (NHS) Contact Tracing platform. Through a shared platform, the Contact Tracing Center could be granted access to a limited data set or escalate reported contacts to the NHS Contact Tracing system. The contact tracing team liaises with index cases for the reporting of any additional contacts and calls all listed contacts for quarantine advice. Based on the data collected, the tier management team and environmental health officers work in synergy with local councils toward local containment strategies, similar to how the local outbreak in Leicester was managed. Figure 2 shows the workflow of the proposed intervention.

## Results

### Search Results

**Figure 3:**
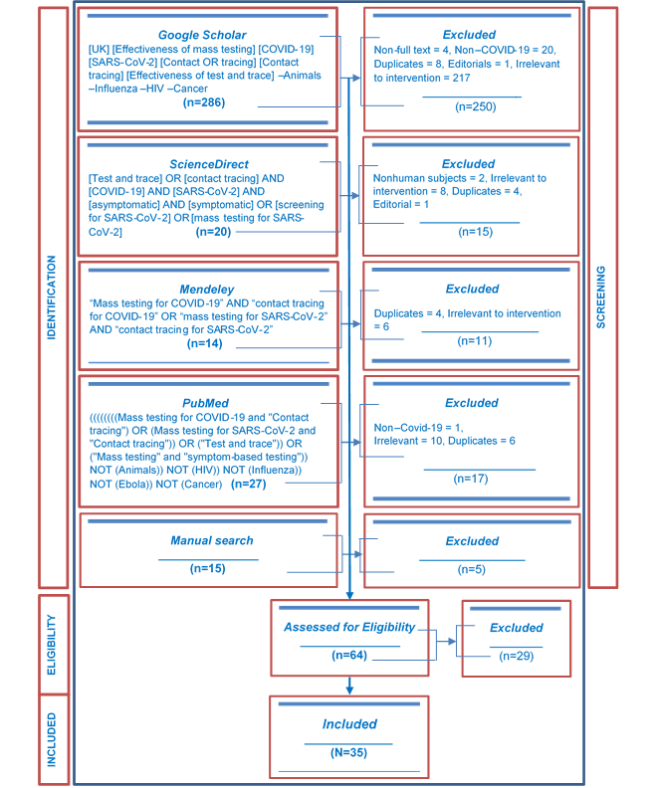
Flowchart showing article counts at each stage as well as the number of included articles.

The search yielded 286 articles from Google Scholar, 20 articles from ScienceDirect, 14 articles from Mendeley, 27 articles from PubMed, and 15 articles from other sources, for a total of 362 articles. Altogether 64 eligible articles were screened for inclusion. Given the ambiguity in the use of contact tracing in most studies to include testing, studies evaluating the effectiveness of contact tracing were included, provided they had a component of mass testing. Considering the novelty of the term “test and trace” used in this study, it is commonplace to find contact tracing based on symptom testing used in studies to be likened to test and trace in this review. A total of 35 articles that met the eligibility criteria were included in the review. A flowchart of how articles were selected can be seen in Figure 3.

Table 1 shows a brief description of the included studies [44-78]. Detailed characteristics of the studies can be found in Table S1 of Multimedia Appendix 1. Table S1 of Multimedia Appendix 2 presents the characteristics of excluded studies [79-107].

**Table 1:** Summary description of included studies.

Study		Description
**Effectiveness**		
	Emery et al [44]	Asymptomatic transmissions among 3711 cruise ship passengers and crew, Japan
	Grassly et al [45]	Percent reduction in reproduction number (hypothetical sample), United Kingdom
	Tsou et al [46]	Outbreak containment using 393 COVID-19 cases, Taiwan
	Mizumoto et al [47]	Asymptomatic cases among 3063 cruise ship passengers, Japan
	Sasmita et al [48]	Infections using COVID-19 data, Indonesia
	Moghadas et al [49]	A hypothetical population of 10,000 to measure required isolation and curtail silent transmission, Canada
	Bracis et al [50]	SARS-CoV-2 transmissions projection using daily COVID-19 cases of King County from March 8-29, United States
	Pollmann et al [51]	Impact of digital contact tracing (hypothetical sample)
	Hill et al [52]	Reduction in infections using contact data from 2010, United Kingdom
	Gorji et al [53]	Reduction in reproduction number (hypothetical sample), Switzerland
	Alsing et al [54]	Intervention efficacy using commuter data from 2011, United Kingdom
	Hagan et al [55]	SARS-CoV-2 prevalence among incarcerated persons in 6 jurisdictions, United States
**Cost-effectiveness**		
	Paltiel et al [56]	Evaluate clinical and economic performance using a hypothetical cohort of 4990, United States
**Asymptomatic proportion**		
	Porru et al [57]	Health surveillance among 5942 staff of a hospital, Italy
	Nishiura et al [58]	Asymptomatic ratio among 565 passengers, Japan
	Treibel et al [59]	Asymptomatic carriers among 400 health care staff, United Kingdom
	Abeysuriya et al [60]	SARS-CoV-2 prevalence among 180 pregnant women, United Kingdom
	Brown et al [61]	SARS-CoV-2 prevalence among 1152 health care workers in 6 hospitals, United Kingdom
	Graham et al [62]	Infections, clinical features, and outcome among 464 residents and staff in care homes, United Kingdom
	Arons et al [63]	Transmission and adequacy of symptom-based screening among 89 residents of a skilled nursing home, United States
	Jameson et al [64]	Asymptomatic infections among 121 nonsymptomatic health care staff, United States
	Callaghan et al [65]	Prevention effectiveness and prevalence of SARS-CoV-2 among 46 patients and 171 health care staff, United States
	Louie et al [66]	Transmission monitoring among 734 persons, United States
	Gudbjartsson et al [67]	Transmissions among 9199 targeted, 10,797 openly invited, and 2283 randomly sampled persons, Iceland
	Reid et al [68]	Testing and cases among 5204 health care staff, Canada
	Lavezzo et al [69]	Population exposure among 2812 residents before and 2343 residents after the lockdown, Italy
	Kimball et al [70]	The utility of symptom screening among 76 older adults in a skilled nursing home, United States
	Olalla et al [71]	Asymptomatic cases among 498 health care staff, Spain
	Guery et al [72]	Infections among 136 nursing care home staff, France
	Roxby et al [73]	COVID-19 morbidity among 142 staff and residents in a residential community, United States
	Lytras et al [74]	SARS-CoV-2 prevalence among passengers repatriated from the United Kingdom (n=357), Spain (n=394), and Turkey (n=32) to Greece
	Hoehl et al [75]	Infections among 125 passengers evacuated to Germany
	Cao et al [76]	Prevalence among 9,899,828 residents in China
	Baggett et al [77]	Infections among 408 homeless shelter residents, United States
	Imbert et al [78]	Infections among 150 homeless shelter residents, United States

Of the 35 studies, 12 (34%) were models, 1 (3%) was a cohort study, and 22 (63%) were cross-sectional studies. In total, 11 studies were implemented in the United States [50,55,56,63-66,70,73,77,78], comprising a sample population of 23,088 participants. Of the 35 studies, 7 (20%) were implemented in the United Kingdom [45,52,54,59-62], with a sample size of 2196 in addition to the real-world data sets that were used in the modeling studies. Three of the studies (8%) were implemented in Japan [44,47,58], with a sample size of 7339. Two of the studies (6%) were implemented in Canada [49,68], with an overall sample size of 5204 subjects (one of the studies used a hypothetical sample). Two studies (6%) were implemented in Italy [57,69], with an overall sample of 11,097 subjects. One study (3%) was implemented in each of the following countries: Taiwan (n=393 subjects) [46], Indonesia [48] using COVID-19 data, Switzerland [53], Spain (n=498 subjects) [71], Germany (n=125 subjects) [75], Greece (n=783 subjects) [74], France (n=136 subjects) [72], Iceland (n=22,297 subjects) [67], and China (n=9,899,828 subjects) [76]. The studies by Moghadas et al [49], Pollmann et al [51], Hill et al [52], and Paltiel et al [56] made use of hypothetical samples.

### Methodological and Risk of Bias Assessment

The methodology and risk of bias assessment was organized according to study design and using the most comprehensive assessment tools. This review made use of the “whole study” assessment method and deployed study design–specific tools, due to the lack of a standardized tool for nonrandomized controlled studies [33,108]. This review’s critical appraisal is also in line with the PHO MetQAT 1.0 quality appraisal tool [32].

#### Modeling Studies

The Relevance and Credibility Assessment for Modeling Studies tool was used to evaluate the methodology and risk of bias of modeling studies [34]. A total of 12 (34%) modeling studies [44-54,56] were included and assessed for risk of bias. Of the 12 studies, 5 (42%) were judged to be at low risk of bias, 4 (33%) to be at moderate risk of bias, and 3 (25%) to be at high risk of bias. The main concerns regarding the risk of bias included inappropriate population and setting: no real-world data set leading to either an unreported or inadequately reported validation process of models. There were issues with either the model validation process or the use of a real-world data set across 7 of the 12 studies (58%) that were rated to be either at moderate or at high risk of bias. Above all, the models were based on a series of assumptions, most of which may not work in real life. A summary of the risk of bias assessment of modeling studies is presented in Table 2. A more detailed risk of bias assessment of models can be found in Table S1 of Multimedia Appendix 3.

**Table 2:** Risk of bias of modeling studies.

Study		Relevance	Credibility	Overall risk
**Effectiveness**				
	Emery et al [44]	Insufficient	Insufficient	Low
	Grassly et al [45]	Sufficient	Sufficient	Low
	Tsou et al [46]	Insufficient	Insufficient	High
	Mizumoto et al [47]	Insufficient	Insufficient	Moderate
	Sasmita et al [48]	Insufficient	Insufficient	Moderate
	Moghadas et al [49]	Sufficient	Insufficient	High
	Bracis et al [50]	Insufficient	Sufficient	Low
	Pollmann et al [51]	Insufficient	Insufficient	High
	Hill et al [52]	Sufficient	Sufficient	Low
	Gorji et al [53]	Insufficient	Insufficient	Moderate
	Alsing et al [54]	Sufficient	Insufficient	Low
**Cost-effectiveness**				
	Paltiel et al [56]	Insufficient	Insufficient	Moderate

#### Cohort Study

The single cohort study [57] included in the review was rated to be at moderate risk of bias, principally due to unsuitable population and setting. This study was implemented in Italy. The study’s risk of bias was assessed using the CASP checklist for cohort studies [35]. In this study, contact tracing was limited to control. There could have been issues surrounding participant selection due to unreported eligibility criteria. In addition, no details were provided about loss to follow-up and how this was managed. Table S1 of Multimedia Appendix 4 provides a detailed risk of bias assessment for this study.

#### Cross-sectional Studies

The risk of bias assessment of cross-sectional studies was conducted using the SURE tool [36]. A total of 22 cross-sectional studies were assessed: 5 (23%) were judged to be at low risk of bias, 1 (4%) at moderate risk of bias, and 16 (73%) to be at high risk of bias. The authors of 10 (45%) studies failed to clearly state their study design. The study population and setting were unrepresentative in up to 82% (n=18) of the studies. Contact tracing as part of the intervention was lacking in 27% (n=6) of studies. The authors in 15 of the 22 studies (68%) did not justify their sample size. The fair selection of participants was not clear in 73% (n=16) of studies due to unreported eligibility criteria. Statistical methods used in study analysis were unreported in 45% (n=10) of studies, while the reporting of statistical analysis was judged to be inadequate in 18% (n=4) of studies. Nine studies (41%) did not provide technical details regarding sample collection and management. Additionally, only 50% (n=11) of studies provided technical details about testing. Unreported blinding was observed in 95% (n=21) of studies. Seven studies (32%) did not report limitations, leading to possible study bias. Lack of participant characteristics was also observed in 32% (n=7) of studies. Bias due to conflicting interests was judged to be possible in 18% (n=4) of studies since the authors’ conflicts of interest were not declared. Table 3 displays a summary of the risk of bias rating for cross-sectional studies. A detailed examination of how cross-sectional studies were assessed is found in Table S1 of Multimedia Appendix 5.

**Table 3:** Risk of bias of cross-sectional studies.

Study		Overall risk
**Effectiveness**		
	Hagan et al [55]	High
**Asymptomatic proportion**		
	Nishiura et al [58]	High
	Treibel et al [59]	High
	Brown et al [61]	Low
	Graham et al [62]	Low
	Abeysuriya et al [60]	Low
	Arons et al [63]	High
	Jameson et al [64]	High
	Callaghan et al [65]	High
	Louie et al [66]	Moderate
	Gudbjartsson et al [67]	High
	Reid et al [68]	High
	Lavezzo et al [69]	Low
	Kimball et al [70]	High
	Olalla et al [71]	High
	Guery et al [72]	High
	Roxby et al [73]	High
	Lytras et al [74]	High
	Hoehl et al [75]	High
	Cao et al [76]	Low
	Baggett et al [77]	High
	Imbert et al [78]	High

### Synthesis of Results

#### Is There Evidence That Mass Testing and Contact Tracing Could Suppress the Community Spread of SARS-CoV-2 Infections Better Than Test and Trace?

Vote counting was deployed as the method to synthesize results, in line with the direction of effect that was used. Studies were prioritized based on their degree of bias in the reported evidence. The GRADE diagram for assessing the quality of evidence was used to grade the evidence presented by the different studies [109].

#### Effectiveness

Of the 12 studies categorized under this outcome, 4 (33%) were at high risk of bias, 3 (25%) were at moderate risk of bias, and 5 (42%) were rated as low. A total of 9 (75%) studies [44,46,47,49,51-55] were voted in favor of the intervention (95% binomial exact [BE] CI 42.8%-94.5%, *P*=.15). Three of the 12 (25%) studies [45,48,50] showed an unfavorable direction of effect and were voted in favor of the control (95% BE CI 5.5%-57.1%, *P*=.15). The body of evidence presented by the 11 modeling studies [44-54] for this outcome was downgraded by three levels to “very low.” First, studies were downgraded one level because they were neither randomized controlled trials nor real-time studies. An additional two levels of downgrading were due to serious study bias, interstudy variation, imprecision, and indirectness. The evidence from the lone cross-sectional study by Hagan et al [55] was downgraded by three levels to “very low” as well. It was downgraded by one level because the study was not a randomized controlled trial and was further downgraded by two levels due to methodological issues, imprecision, and indirectness.

#### Cost-effectiveness

The single study found for this outcome [56] was voted in favor of the intervention. This study was judged to be at high risk of bias. The quality of evidence was downgraded by one level given that it was not a randomized controlled trial. Being a model based on assumptions, coupled with study limitations, imprecision, and indirectness, the evidence was further downgraded by two levels. The evidence was classified as very low.

#### Safety

We found no study addressing this outcome. There have been mixed views regarding the safety of mass testing and contact tracing. Some argue that rapid mass testing will lead to false positives and negatives, thereby causing misinformation [79,110]. Others see both rapid mass testing and contact tracing as safety nets against virus spread [111-114]. Both nasopharyngeal and oropharyngeal swaps appear to be slightly invasive. There also exists a body of evidence regarding safety and security concerns from the public on contact tracing [115-117].

#### Acceptability

Again, no study was found regarding this outcome. Altmann and colleagues [111] found a high level of acceptance for app-based contact tracing. Their investigation was done across different countries including the United Kingdom [111]. It was also reported that there is a higher preference for government contact tracing applications than those managed by private companies [22].

#### Equity

There was no study evaluating this outcome. It remains, however, clear that the test and trace system is not equitable [18]. Testing that is delivered near the patient and at a walkable distance increases equity [118,119].

#### Binomial Test and 95% CI

A total of 13 studies were retained to assess the primary objective. Statistical synthesis for the primary objective was based on the binomial probability test and BE CIs performed in Stata 14.2 (StataCorp LLC). Of the 13 studies, 10 (76.9%) favored the intervention (95% BE CI 46.2%-95.0%, *P*=.09), with just 3 (23%) studies voted in favor of the control (95% BE CI 5%-54%, *P*=.09). The above indicates that the intervention is a better strategy than the control in the suppression of SARS-CoV-2 transmissions. The probability that the above estimate is true if the conventional Test and Trace program was truly better than mass testing and contact tracing is just 9%. The 76.9% (10/13) favorable direction of effect is a clear enough majority vote to indicate that mass test and trace is truly more beneficial.

**Figure 4:**
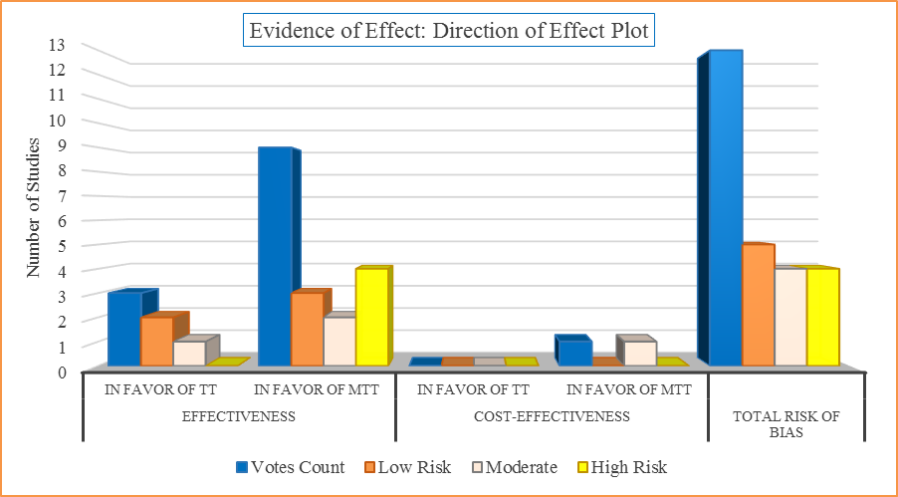
Evidence of effect attributable to the intervention (mass testing and contact tracing, MTT) and control (test and trace, TT) for the primary objective.

Assuming that the true probability of both mass testing with contact tracing and test and trace being equivocal is .50 under the null hypothesis (H_0_: mass test and trace=test and trace), this study observed 10 out of 13 votes (76.9%), which is well above the expected binomial probability mean of 6.5 (SD 1.803) votes. Of the 10 studies, 4 (40%) in favor of the intervention were judged to be at high risk of bias, 3 (30%) at moderate risk of bias, and 3 (30%) at low risk of bias. A total of 23% (n=3) of the retained studies had representative samples and settings. Two of 3 studies (67%) implemented in the United Kingdom [52,54] voted in favor of the intervention were judged to be at low risk of bias. The effect direction plot of different studies, together with the associated risk of bias, is shown in Figure 4.

The results of 6 studies [44,47,52-54,56] were judged to be at low to moderate risk of bias. These studies were prioritized in concluding that the mass testing and contact tracing strategy was more effective in the suppression of community transmission of SARS-CoV-2 and the control of COVID-19 than conventional test and trace. The studies by Emery et al [44], Hill et al [52], and Alsing et al [54] were judged to be at low risk of bias. Two of these (ie, [52,54]) were both representative of the population and evaluated mass testing and contact tracing as a hybrid strategy, in line with the primary objective. Emery et al [44] failed to consider contact tracing but compared the effectiveness of testing based on symptoms and testing irrespective of symptoms. We concluded that the direction of effect will not be different if contact tracing were to be integrated since contact tracing is contingent on testing.

The generated GRADE evidence profile was used to present the synthesis findings regarding the primary objective (Table 4). Table S1 of Multimedia Appendix 6 provides details of how the evidence for different outcomes under the primary objective was graded.

**Table 4:** Grading of Recommendations Assessment, Development and Evaluation (GRADE) evidence profile: certainty of evidence for the primary objective.

Outcome		Studies, n	Quality of evidence factors					Direction of effect SOF^a^			Quality of evidence^b^
			Limitation	Heterogeneity	Indirectness	Imprecision	Publication bias	TT^c^, n	MTT^d^, n	Direction^e^	
**Effectiveness**											
	Model	11	Serious	Serious	Serious	Serious	Unlikely	3	8	↑	Very low
	Cross-sectional study	1	Not serious	Unlikely	Serious	Serious	Unlikely	0	1	↑	Very low
**Cost-effectiveness**											
	Model	1	Serious	Unlikely	Serious	Serious	Unlikely	0	1	↑	Very low

^a^SOF: summary of findings.

^b^Quality of evidence graded as either “very low,” “low,” “moderate,” or “high.”

^c^TT: test and trace.

^d^MTT: mass testing and contact tracing.

^e^↑MTT is better than TT; ↓TT is better than MTT; ↔ MTT and TT are equivocal.

#### What Is the Proportion of Asymptomatic Cases of SARS-CoV-2 Reported During Mass Testing Interventions?

**Figure 5:**
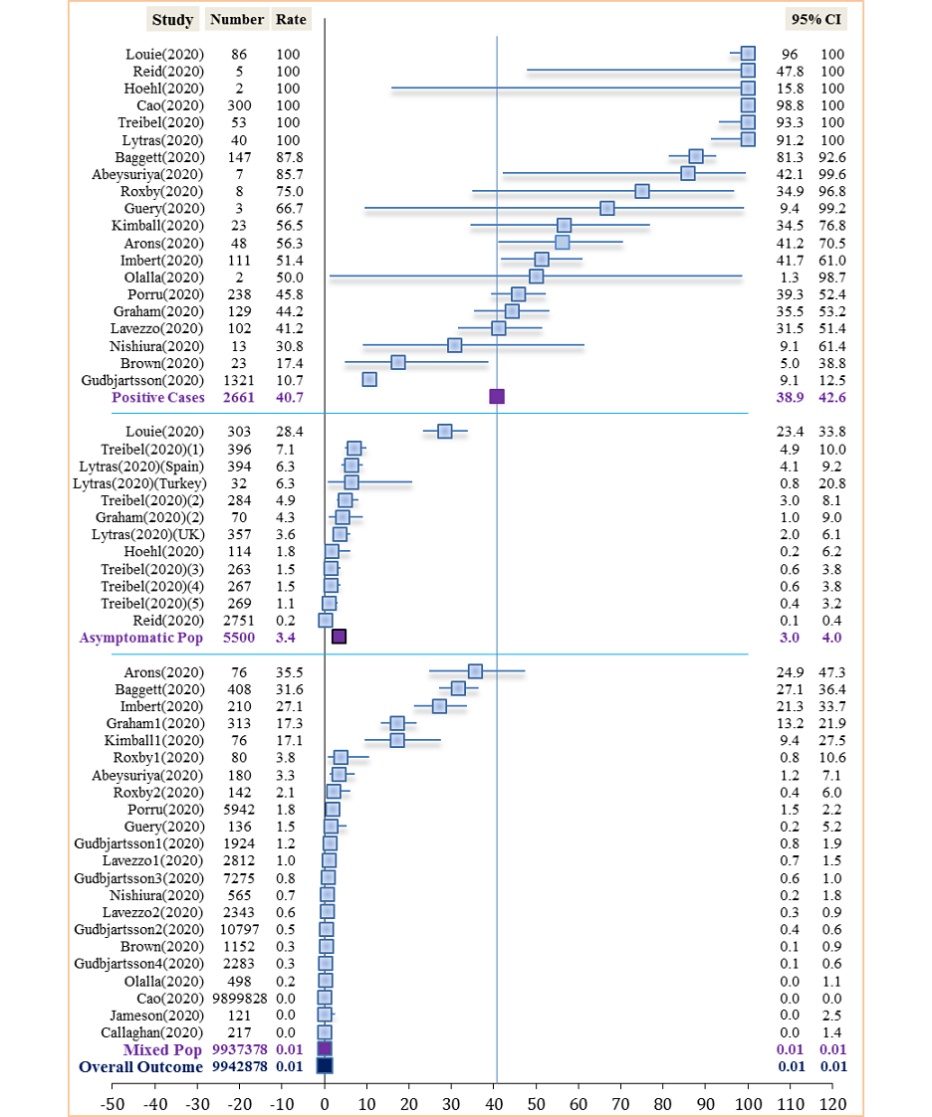
Asymptomatic SARS-CoV-2 carriers among detected cases, in asymptomatic and mixed-sample populations.

A total of 21 cross-sectional studies and 1 cohort study [57-78] were retained under the secondary objective. There was limited precision in effect estimates with just 27% (6/22) of studies providing data on CIs for the proportion of asymptomatic carriers. Of the 22 studies, 7 (32%) were judged to be at low to moderate risk of bias. A graphical presentation of the asymptomatic proportion from the 22 studies (34 reports) can be seen in Figure 5. The sampled population ranged from 76 to 9,899,828 subjects, with a median sample of 395.5 subjects. The number of detected positive SARS-CoV-2 cases and asymptomatic carriers ranged from 0 to 1321 and from 0 to 300, respectively. Likewise, the mean number of positive cases and asymptomatic carriers were 120.9 (SD 280) and 49.3 (SD 71.1), respectively.

#### Outcome Among Stratified Positive Cases

**Figure 6:**
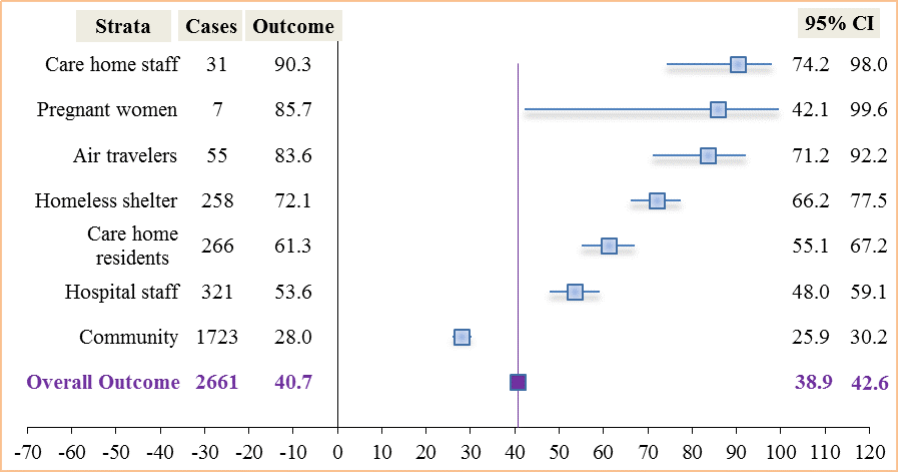
Asymptomatic SARS-CoV-2 carriers among stratified positive cases.

The proportion of asymptomatic cases among those testing positive ranged from 28% (483/1723, 95% CI 25.9%-30.2%) in the community (testing of residents) to 90.3% (28/31, 95% CI 74.2%-98.0%) among care home staff. The overall proportion was found to be 40.7% (1084/2661, 95% CI 38.9%-42.6%) (Figure 6). Two studies [64,65] with sample sizes of 121 and 217 subjects, respectively, detected neither cases nor found any asymptomatic carriers and were excluded in the evaluation of asymptomatic carriers among persons who tested positive.

#### Outcome Among Stratified Sample Populations

**Figure 7:**
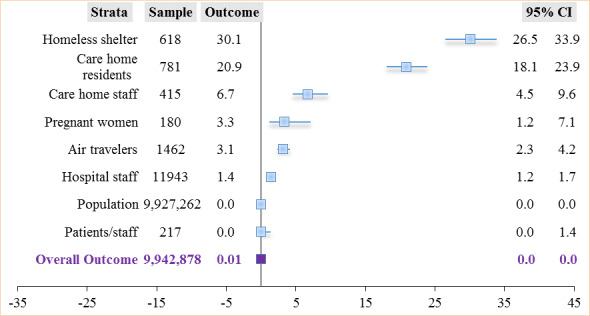
Asymptomatic SARS-CoV-2 carriers in the stratified overall sampled population.

The prevalence of asymptomatic SARS-CoV-2 cases was highest among homeless shelter residents (186/618, 30.1%; 95% CI 26.5%-33.9%), followed by care home residents (163/781, 21%; 95% CI 18%-24%), and lowest among hospital patients (0/217, 0%; 95% CI 0.0%-1.4%). The overall prevalence for all studies was 0.01% (1084/9,942,878; 95% CI 0.0%-0.0%). Excluding screening in the general population in the studies by Cao et al [76], Gudbjartsson et al [67], and Lavezzo et al [69], overall asymptomatic SARS-CoV-2 prevalence for all other settings was found to be 3.8% (601/15,616, 95% CI 3.5%-4.2%). Figure 7 shows the outcome prevalence in various specific sample populations.

The prevalence among asymptomatic populations from 6 studies [59,62,66,68,74,75] was 3.4% (189/5500, 95% CI 3%-4%). The prevalence in a mixed population from 17 studies [57,58,60-65,67,69-73,76-78] averaged 0.009% (895/9,937,378, 95% CI 0.0%-0.0%) (Figure 5).

#### Outcome Within the United Kingdom

**Figure 8:**
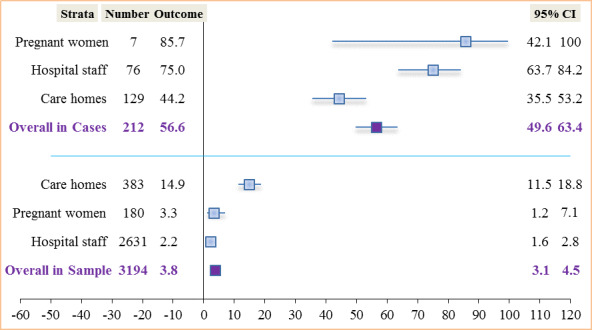
Asymptomatic SARS-CoV-2 carriers among cases and in the sampled population in the United Kingdom.

Four studies [59-62] evaluated the outcome within the United Kingdom. Treibel et al [59] and Brown et al [61] evaluated the outcome among hospital staff, Graham et al [62] evaluated it in care homes, and Abeysuriya et al [60] among pregnant women at term. The overall asymptomatic SARS-CoV-2 proportion among detected cases in the United Kingdom was found to be 56.6% (120/212; 95% CI 49.6%-63.4%). The proportion of asymptomatic cases among those tested positive ranged from 44.2% (57/129; 95% CI 35.4%-53.2%) in care homes to 85.7% (6/7; 95% CI 42.1%-100%) in pregnancy. Figure 8 shows the relationship of asymptomatic proportion among detected cases and in the sampled population in different settings within the United Kingdom.

The overall prevalence of asymptomatic cases within the United Kingdom was found to be 3.8% (120/3194; 95% CI 3.1%-4.5%) with rates ranging from 2.2% (57/2631; 95% CI 1.6%-2.8%) among hospital staff to 14.9% (57/383; 95% CI 11.5%-18.8%) in care homes. Figure 8 demonstrates a higher overall rate among detected cases in the United Kingdom (120/212, 56.6%) compared to that of all studies (z=4.52, *P*<.001). We found in this review that asymptomatic cases were 1.4 times (56.6%/40.7%) more likely to be detected among positive cases in the United Kingdom than all studies put together. The overall SARS-CoV-2 prevalence rate in the United Kingdom (120/3194, 3.8%) was similar to that of all studies put together (601/15,616, 3.8%), excluding studies undertaken at the population level.

All unreported and unsuitable CIs were generated in Stata 14.2 (BE) and exported to Excel. The rule of three was applied to the studies by Jameson et al [64] and Callaghan et al [65] due to zero-outcome events in their sampled populations.

### Interstudy Variability

Variations among studies included in the primary objective were mainly due to the study population and setting, assumptions, and model structure. We observed that only 3 of 13 studies (23%) synthesized under the primary objective were representative of the population. Apart from deploying different model types, some studies made use of real-time COVID-19 data sets, whereas others used historic data sets or relied on hypothetical samples. This increased variability and reduced the generalizability of the results. However, 2 of the 3 (66.7%) studies implemented in the United Kingdom were in favor of the intervention.

An observation of plotted graphs under the secondary objective showed high heterogeneity when measuring the outcome among detected SARS-CoV-2 cases, mainly due to methodology (Figure 5). Some studies were implemented at the population level while others purposefully used asymptomatic populations. Additionally, a limited number of studies provided details on the type of test used as well as how test samples were managed (Table S1, Multimedia Appendix 7). However, there was observed minimal heterogeneity among studies when stratified, mostly stemming from the study implemented among pregnant women; this was a single study by Abeysuriya et al [60], with a small sample of 180 pregnant women at term. The median age of these women was just 29.9 years (SD 7.4). This is contrary to the belief that infections are more prevalent in older populations. A stratification of the different studies by setting produced similar rates for studies implemented in the United Kingdom and all studies pooled together, excluding population-level studies. Excluding the largest citywide study (n=9,899,828 subjects) [76] from this review increased the overall SARS-CoV-2 prevalence in the sampled population to 1.8% (784/43,050; 95% CI 1.7%-1.9%).

## Discussion

### Principal Findings

Although considered low-level evidence, our review synthesis has shown a clear majority vote of 76.9% (10/13; 95% BE CI 46.2%-95.0%, *P*=.09) in favor of mass testing and contact tracing.

We also found an overall proportion of asymptomatic carriers among detected positive cases to be 40.7% (1084/2661; 95% CI 38.9%-42.6%) for all studies, compared to 56.6% (120/212; 95% CI 49.6%-63.4%) within the United Kingdom when stratified. The proportion of asymptomatic cases across studies ranged from 28% (483/1723) among cases detected in the general population to 90% (28/31) among care home staff with positive tests. In addition, asymptomatic SARS-CoV-2 prevalence was highest among residents in homeless shelters (186/618, 30.1%) and lowest among hospital patients (0/217, 0.0%). The overall prevalence of asymptomatic cases in the sampled population was 0.01% (1084/9,942,878; 95% CI 0.0%-0.0%) compared to 3.8% (120/3194; 95% CI 3.1%-4.5%) within the United Kingdom.

### Comparison With Prior Work

Studies that were in favor of the control in this review assumed that mass testing was not feasible, as acknowledged by Peto [80]. Evidence from countries that embarked on mass testing, including Taiwan, Germany, Ireland, China, and India, suggests that regular mass testing and contact tracing could be a game changer. The analysis by Peto et al [80, 112] showed that mass testing and contact tracing is by far more cost-effective than the present test and trace method, which is in line with the second outcome. Maslov [79] shares an opposing view in that even the slightest false positives will render random mass testing an unreliable policy. While Maslov [79] seems to be concerned with the inherent moral decadence of unjust isolation, it is better to be on the safe side than to be amid false negatives and contented asymptomatic carriers. Symptomless testing to identify asymptomatic carriers is crucial because Viswanathan and colleagues [10] also acknowledged that strategies based on symptom screening could miss between 40%-100% of infected persons. A study among pregnant women at term in East London by Abeysuriya et al [60] found the sensitivity of testing based on symptoms to be as low as 14.3% (95% CI 0.36%-57.87%). Paying attention to asymptomatic infections as cases that could be missed has also been underscored by Byambasuren et al [120]. This is concordant with the key messages and objectives of the European Centre for Disease Prevention and Control that countries should test the whole population in high-transmission settings [121].

The 40.7% (1084/2661) asymptomatic proportion among positive cases found in this review is in line with the 40%-45% proportion estimated by Oran and Topol [122]. Clarke and colleagues [123] reported a similar rate of 40.3% among hemodialysis patients. This proportion is also similar to that reported in Spain (40.5%) by Albalate and colleagues [124]. The asymptomatic proportion among detected positive air travelers (46/55, 83.6%) we found in this review is higher than the 76.6% reported by Al-Qahtani et al [125], perhaps due to more awareness as the study was implemented at a much later date. Yanes-Lane et al [126] reported an asymptomatic proportion of positive cases among care home residents (54%), which is just slightly lower than the 61.3% (163/266) reported in this review. Notwithstanding the overarching reported high infectivity from asymptomatic individuals, we report rates in this review ranging from 0.003% (300/9,899,828) to 1.2% (24/1924) in the population. This is contrary to the rates (1.5%-2.8%) reported by Wu and McGoogan [127]; this higher rate could have been because testing was initially done among symptomatic individuals since asymptomatic proportions normally remain higher among index cases. In this review, we estimated that the proportion of asymptomatic SARS-CoV-2 carriers among cases in the general population was 28% (483/1723) (Figure 6), in agreement with the community asymptomatic proportion of 28% reported in Beale et al [128]. In contrast, Petersen and colleagues [129] reported a community asymptomatic proportion that was 3 times higher (76.5%-86.1%). This population-level study was undertaken in the United Kingdom, contrary to those included in this review that were conducted in Iceland, Italy, and China. The largest population sample in this review, from Cao et al [76], was a study done immediately after the lockdown, which could be the reason behind the low rate of asymptomatic cases.

### Limitations

A substantial number of included studies were models, which normally rely on assumptions that may not be achieved in real life. Expert knowledge was needed to evaluate the validation process of models. This might have affected the results. The fact that this review went through a single reviewer could have introduced some bias in study selection and analysis. The variability in the understanding of mass testing by different researchers might have affected the analysis as well. In addition, review results could have been affected by differences in sample handling and testing methods, coupled with the lack of provision of technical details about testing. This review was language biased since the literature search was limited to English articles. This review was not registered with PROSPERO (International Prospective Register of Systematic Reviews) per standard systematic review practice.

### Public Health Implications

Controlling a virus whose manifestation changes over time and increasingly without signs is not about the number of tests but about who needs to be tested. The pertinent questions relate to when people should be tested, where they should be tested, and how often. An appropriate public health strategy that will get the right people tested, at the right time, in the right place, and at regular intervals requires a community-based and participatory approach that will not be without a greater cost burden. At the center of such a strategy is overcoming the challenges related to the scarcity of supplies and waiting time, through the development of rapid tests [130]. Among others, winning public confidence; ensuring data security, acceptability of the contact tracing apps, and equity of testing and contact tracing; use of rapid tests; capacity building and system strengthening; effective monitoring of isolation/quarantine and program sustainability are some factors to be considered. More real-time research is needed regarding the effectiveness of mass testing and contact tracing to obtain a better picture of disease burden and mitigation strategies.

### Conclusions

We sought to critically evaluate the evidence that mass testing and contact tracing is a better strategy for controlling local transmissions of SARS-CoV-2 in the United Kingdom compared to the conventional test and trace method. We have demonstrated a very low level of promising evidence that mass testing and contact tracing could be more effective in bringing the virus under control and even more effective if combined with social distancing and face coverings. The implementation of test and trace should be done at mass irrespective of symptoms with the local community, through GP surgeries, community health centers, and local councils [131]. The proposal is for the present Test and Trace program to be superseded by a decentralized and continuous mass testing program with rapid tests, championed by community services with low resource needs [81]. The following recommendations could therefore be useful:

- Capacitate GP surgeries and community health services to deliver mass testing at the point of care [132];
- The government should work in synergy with local councils for surveillance, isolation, and quarantine [132]. This resulted in major success in Germany [133,134];
- Regular organizational and company-wide testing for the safe resumption of economic activities [135];
- Testing should be a border control measure for all travelers [82,83];
- Testing of prisoners, detainees, and all those in congested accommodations [49]. A good example is the Lesbos camp testing [136,137];
- Sewage and environmental testing should be part of mitigation strategies.

## Appendix

Multimedia Appendix 1 Characteristics of included studies.

Multimedia Appendix 2 Characteristics of excluded studies.

Multimedia Appendix 3 Quality assessment of modeling studies.

Multimedia Appendix 4 Quality assessment of the cohort study.

Multimedia Appendix 5 Quality assessment of cross-sectional studies.

Multimedia Appendix 6 Certainty of evidence for the primary objective.

Multimedia Appendix 7 Details of mass testing.

## Abbreviations

BE: binomial exact
CASP: Critical Appraisal Skills Program
GP: general practitioner
GRADE: Grading Recommendations Assessment, Development, and Evaluation
MetQAT: Meta Quality Appraisal Tool
NHS: National Health Service
PROSPERO: International Prospective Register of Systematic Reviews
SURE: Specialist Unit for Review Evidence
SWiM: Synthesis Without Meta-Analysis
