# Supplementary material for: Mass Testing With Contact Tracing Compared to Test and Trace for the Effective Suppression of COVID-19 in the United Kingdom: Systematic Review
Source: JMIRx Med. 2021 Apr 12;2(2):e27254. doi: 10.2196/27254 (PMC8045129; doi:10.2196/27254)
Supplement: Multimedia Appendix 1 [file xmed_v2i2e27254_app1.pdf]

Table S1: Characteristics of Included Studies

Study	Design/ setting	Sample	Strategies	Assumptions	Main findings
**Effectiveness**					
Emery et al [44]	• Modeling study in Japan	• 3711	• Symptom-based testing<br>• Symptom-agnostic testing	• Constant infectiousness<br>• Progress to presymptomatic/ asymptomatic is irrespective of the origin of infections.<br>• Unavailable symptom onset date for 115 cases proportional to cases with reported dates<br>• Unavailable test dates for 13 persons proportionate to tests among those with unreported symptom onset.<br>• Proportion of asymptomatic infectiousness<br>• Individual test negative after the infectious period<br>• Test accuracy = 100%<br>• People are 50% more likely to be tested in biased symptom-agnostic testing	• Testing irrespective of symptoms showed to be more effective in case identification than symptom-based testing
Grassly et al [45]	• Modeling study in the UK	• Hypothetical	• Symptom-based self-isolation<br>• Symptom testing and case isolation.	• Asymptomatic individuals are less infectious than symptomatic individuals.<br>• 100% Polymerase chain	• Self-isolation upon symptom onset will reduce transmissions by 47% (95% Uncertainty

Study	Design/ setting	Sample	Strategies	Assumptions	Main findings
			• Regular testing of high-risk groups irrespective of symptoms & isolation<br>• Test and trace of contacts & isolation<br>• Contact tracing by symptoms alone.<br>• Test-trace-test contacts and isolate	reaction (PCR) test sensitivity<br>• 100% coverage of Test and Trace<br>• Sample collection is done at symptom onset.<br>• 1 day delay from sample collection and quarantining of contacts.<br>• 80% of symptomatic cases are reported.<br>• 80% of symptomatic contacts are traced.<br>• Testing is done on the day of symptom onset	Interval, UI: 32-55).<br>• Screening all healthcare workers and other high at-risk populations every week will further reduce transmission by 23% (95% Uncertainty Interval: UI 16–40) in addition to that achieved by isolation.<br>• Test and trace will further reduce transmissions by 26% (95% UI:14-35)
Tsou et al [46]	• Modeling study in Taiwan	• 393	• Symptom-based testing and isolation of index cases<br>• Mass testing of symptomatic and asymptomatic subclinical cases<br>• Symptom-based testing, isolation, and quarantine of all at-risk group	• Incubation period per case and symptom onset to isolation delay, follow a Weibull distribution.<br>• Potential secondary cases follow a negative binomial distribution with mean = reproduction number R<br>• Strategies differed in their control of subclinical cases.<br>• Initial number of cases = 5, 20 & 40<br>• At-risk persons investigated = 40%, 60%, 80% & 90%	• The strategy of symptom-based testing, isolation, and quarantining all subclinical cases was most effective.<br>• Strategy B was better than A in the prevention of transmissions before symptom onset

Study	Design/ setting	Sample	Strategies	Assumptions	Main findings
				• 40%, 60%, 80% of subclinical cases assumed to be detected and isolated.<br>• Subclinical cases can completely be prevented	
Mizumoto et al [47]	• Modeling study in Japan	• 3063	• Mass testing	N/A	• A total of 634 cases were detected 328 of whom were asymptomatic.<br>• The proportion of asymptomatic increased over the weeks
Sasmita et al [48]	• Modeling study in Indonesia	• Daily Covid-19 cases	• Scenario 1 = u1+u4+u5<br>• Scenario 2 = u1+u2+u4+u5<br>• Scenario 3 = u1+u2+u3+u4+u5<br>U1 = Large-scale social restriction; U2 = Contact tracing; U3 = Mass testing; U4 = Case detection and treatment; U5 = Face masks use	• 95% false positive rate from susceptible to exposed.<br>• Possibility of reinfections due to loss of immunity<br>• All parameters were assumed to be positive and constant.<br>• Availability of rapid PCR tests	• COVID-19 cases attained peak for strategy 1, 2, and 3 on 59th, 38th, and 40th day after initial outbreak with 33151, 37908, and 39305 cases, respectively.<br>• The optimal control measure was scenario 2 with (u1), (u2), (u4), and (u5)
Moghadas et al [49]	• Modeling study in Canada	• Hypothetical	• No self-isolation<br>• 100% severe cases self-isolate<br>• 100% symptomatic	• The Proportion of asymptomatic infections is 17.9% and 30.8%	• Isolating all symptomatic will still be inefficient in outbreak control.<br>• Combined with case

Study	Design/ setting	Sample	Strategies	Assumptions	Main findings
			case self-isolate<br>• 100% isolation of symptomatic cases plus detection and isolation of asymptomatic cases		isolation, results indicated that 33% and 42% detection and isolation of silent infections would be needed to suppress the attack rate below 1%, for asymptomatic proportions of 17.9% and 30.8%, respectively
Bracis et al [50]	• Modeling study in the USA	• Daily Covid-19 cases	• No intervention<br>• Isolating the elderly<br>• Schools opening in fall.<br>• Testing, treatment, isolation, and contact tracing in combination with physical distancing	• 20% of infections are symptomatic.<br>• Homogenous infectivity and outcome<br>• Constant diagnostic rate<br>• More than 40% diagnosed during early testing.<br>• 50% of contacts are successfully traced.<br>• Contact tracing permits 5% of asymptomatic and subclinical to be tested.<br>• Differential post-COVID-19 physical interaction	• Ramping up testing, isolation, and contact tracing of symptomatic cases reduced post-COVID interactions by 60% and very few deaths.<br>• Mass testing was not found to be feasible
Pollmann et al [51]	• Modeling study	• Hypothetical	• Digital contact tracing (based on reported symptoms),<br>• Quarantining,	• All contacts using digital contact tracing can be traced.<br>• Unreported symptoms and untested symptomatic cases<br>• Tracing of infected contacts	• Contact tracing must be combined with either random mass testing or social distancing to control the epidemic.

Study	Design/ setting	Sample	Strategies	Assumptions	Main findings
			• Testing<br>• Social distancing<br>• Random testing	• Immediate quarantine upon the report of symptoms<br>• 100% test accuracy<br>• Homogeneous population<br>• Immunity once recovered.<br>• No symptoms-testing delay<br>• Absence of manual tracing<br>• Fixed latent period.<br>• A backward/forward tracing	• Daily random testing of 20% of the population found to be as effective as social distancing
Hill et al [52]	• Modeling study in the UK	• 2010 social contact data	• Test and trace<br>• Regular mass testing	• Each person can be infectious.<br>• Contact network follows the Poisson distribution.<br>• Contact probabilities fall with the level of accommodation.<br>• No random accommodation<br>• People can infect 1-day post symptom onset.<br>• 100% test specificity<br>• Possible to Forget contacts<br>• Self-isolation time=10 days<br>• Test- results delay= 2 days<br>• Contact isolation = 14 days<br>• Adherence to test and trace.<br>• No contacts during isolation<br>• No COVID-19 student beginning the term	• Daily and weekly testing combined with contact tracing adherence reduced the number of infections by more than 50% compared to test and trace alone
Gorji	• Modeli	• Hypo	• Mass testing	• 90% infection reduction due to	• Testing high-risk

Study	Design/ setting	Sample	Strategies	Assumptions	Main findings
et al [53]	ng study in Switzerland	thetical	• Contact tracing.<br>• Smart testing[a] and contact tracing	self-isolation<br>• Basic reproduction number of 2.4 if no mitigation<br>• Test results take 1 day.<br>• Children under 10 contribute little to infections.<br>• The at-risk subpopulation has a 27-fold prevalence rate.<br>• Detection of high contact individuals every 7 days	individuals irrespective of symptoms with contact tracing will reduce R to 1.<br>• Contact tracing based on symptom testing will miss most cases.
Alsing et al [54]	• Modeling study in the UK	• 2011 commuter data and BBC pandemic dataset	• Contact tracing and social distancing.<br>• Contact tracing with Mass testing.<br>• Contact tracing with lockdowns	• Active infections at 8 months<br>• The number of daily tests required.<br>• Effective reproduction number (RE) per scenario<br>• Number of people in lockdown	• Possible to control 38% of outbreak simulations within 8 months using contact tracing with 63.3% of outbreak still leaving R>1.<br>• Mass testing and contact tracing contained 74% of the outbreak simulations with 36.8% of outbreaks resulting in R<1
Hagan et al [55]	• Cross sectional in the USA	• 16161	• Symptom-based testing<br>• Mass testing	N/A	• Mass testing increased the number of COVID-19 cases from 642 (range = 2–181, median = 19) after symptom-based testing to 8,239 (range = 10–2,193, median = 403) giving a

Study	Design/ setting	Sample	Strategies	Assumptions	Main findings
					median increase of 12.3-fold
**Cost-Effectiveness (model)**					
Paltiel et al [56]	• Modeling study in the USA	• 4990 hypothetical cohort	• Base case scenario with a reproduction number (Rt) of 2.5, test specificity of 98%, and 10 new infections each week<br>• Worst case scenario with an Rt of 3.5, test specificity of 98%, and 25 new infections every week<br>• Best case scenario with an Rt of 1.5, test specificity of 99.7%, and 5 new infections each week	• Test frequency = 1, 2, 3 & 7<br>• Test sensitivity = 70%- 99%<br>• Importation of infections via exogenous shocks<br>• Specificity of 98% - 99%<br>• Reproduction number = 1.5, 2.5 and 3.5<br>• Case fatality = 0.05%<br>• 30% chance that infection will lead to virus symptoms.<br>• Cost per test = \$10 - \$50<br>• Abbreviated 80-days period.<br>• A cohort of non-immune students in a congregate setting of 5,000 students<br>• 8-hour test turnaround time<br>• Availability of 100% confirmatory tests at \$100<br>• 25 new cases per week	• A willingness-to-pay of ≤\$5,500/infection averted, screening every week using a 70% sensitive test was optimal.<br>• Regular screening (7, 3 & 2 days) was optimal if only a single test of \$25 with 80% sensitivity was available.<br>• There was no condition under which symptom-based screening alone will contain the outbreak
**Asymptomatic proportion**					
Porru et al [57]	• Cohort study in Italy	• 5942	• Mass RT-PCR[b] testing using oropharyngeal and	• N/A	• A total of 238 cases were detected, of whom 109 were asymptomatic.

Study	Design/ setting	Sample	Strategies	Assumptions	Main findings
			nasopharyngeal swabs		• Mass testing permitted prompt isolation and monitoring of cases
Nishiura et al [58]	• Cross-sectional in Japan	• 565	• RT-PCR testing	• N/A	• 63 passengers were symptomatic.<br>• Four (30.8%, 95% CI: 7.7– 53.8%) of 13 positive cases were asymptomatic and 9 were symptomatic
Treibel et al [59]	• Cross-sectional in the UK	• 396<br>• 284<br>• 263<br>• 267<br>• 269	• PCR test on 400 nasopharyngeal swaps at 5-time points	• N/A	• Twelve (27%) of 44 positive cases were asymptomatic.<br>• Positive. Fifty staff self-isolated as a result of symptoms
Abeysuriya et al [60]	• Cross-sectional in the UK	• 180	• Nasopharyngeal swap PCR test	• N/A	• Seven women tested positive with 6 (85.7 %, 95% CI: 42.1–99.6) as asymptomatic.<br>• Symptom-based testing sensitivity was 14.3% (0.36–57.87) and specificity was 91.86% (86.72–95.48)
Brown et al [61]	• Cross-sectional in the UK	• 1152	• Nasopharyngeal/ oropharyngeal swap PCR tests	• N/A	• Thirteen (57%) of 23 positive cases had symptoms compliant with COVID-19, of whom 4

Study	Design/ setting	Sample	Strategies	Assumptions	Main findings
					(17.4%) were asymptomatic
Graham et al [62]	• Cross-sectional in the UK	• 383	• Comprehensive testing with oropharyngeal and nasopharyngeal swaps<br>• Symptom screening	• N/A	• 126 (40%, 95% CI 35 to 46) of the 313 tested residents were positive.<br>• Only 72 (57%, 95% CI 49–66) positive cases would have been diagnosed based on symptom-testing
Arons et al [63]	• Cross-sectional in the USA	• 76	• Point prevalence testing with RT-PCR on nasopharyngeal and oropharyngeal swabs	• N/A	• 48 (63%) of 76 tested residents were positive of whom 27 (56%) were asymptomatic. 24 of the 27 developed symptoms 1-week post-test
Jameson et al [64]	• Cross-sectional in the USA	• 121	• Universal testing<br>• Universal symptom: screening<br>• Isolation of cases (nasopharyngeal swaps)	• N/A	• No positive case was found among 121 out of 499 eligible healthcare workers screened
Callaghan et al [65]	• Cross-sectional in the USA	• 217	• Nasopharyngeal swap PCR test	• N/A	• No participant tested positive for COVID-19
Louie et al [66]	• Cross-sectional in the	• 303	• Outbreak response mass testing with PCR on	• N/A	• Mass testing identified a high proportion of asymptomatic cases.

Study	Design/ setting	Sample	Strategies	Assumptions	Main findings
	USA		nasopharyngeal swabs		• The symptom-based screening was ineffective in detecting cases among healthcare workers
Gudbjartsson et al [67]	• Cross-sectional in Iceland	• 9199<br>• 10797<br>• 2283	• Targeted testing<br>• Open invitation screening<br>• Random invitation screening<br>(on nasopharyngeal and oropharyngeal samples)	• N/A	• 13.3% tested positive in targeted testing, 0.8% in open invitation testing, and 0.6% in random invitation testing.
Reid et al [68]	• Cross-sectional in Canada	• 2751	• Symptomatic testing<br>• Asymptomatic testing<br>(on nasopharyngeal swabs)	• N/A	• 188 (6.4%) positive cases detected during symptomatic testing and 5 (0.2%) positive cases during asymptomatic testing, with a low probability of testing positive
Lavezzo et al [69]	• Cross-sectional in Italy	• 2812<br>• 2343	• Pre and post-RT-PCR on nasopharyngeal swabs	• N/A	• The first survey gave a prevalence of 2.6% (95% CI: 2.1–3.3%) and 1.2%; 95% CI: 0.8–1.8%) for survey 2. 29 (39.7%; 95% CI: 28.5–51.9%) of positive tests in the survey 1 were asymptomatic and 13 (44.8%; 95% CI: 26.5–

Study	Design/ setting	Sample	Strategies	Assumptions	Main findings
					64.3%) in survey 2.
Kimball et al [70]	• Cross-sectional in the USA	• 76	• RT- PCR mass testing on nasopharyngeal and oropharyngeal swabs	• N/A	• Twenty-three (30%) residents were positive with 13 (57%) either presymptomatic or asymptomatic.<br>• Testing based on symptom screening could miss up to 50% of cases
Olalla et al [71]	• Cross-sectional in Spain	• 498	• Symptom screening<br>• Asymptomatic testing<br>(on nasopharyngeal and oropharyngeal swaps)	• N/A	• 2 asymptomatic on day of sampling tested positive. 1 reported having had symptoms in the last 14 days
Guery et al [72]	• Cross-sectional in France	• 136	• RT-PCR mass testing on nasopharyngeal swap	• N/A	• Three (2.2%) cases detected, 1 of whom was symptomatic and the other developed symptoms within 24 hours
Roxby et al [73]	• Cross-sectional in the USA	• 142<br>• 80	• Repeated RT-PCR mass testing on nasopharyngeal swap (7 days apart)	• N/A	• Five (7%) cases were detected, 3 of which were asymptomatic.<br>• Symptom-based testing might not identify all positive cases
Lytras et al(a) [74]	• Cross-sectional in	• 357	• RT-PCR mass testing using nasopharyngeal	• N/A	• Thirteen (3.6%, CI: 2.0–6.1) positive asymptomatic cases

Study	Design/ setting	Sample	Strategies	Assumptions	Main findings
	Greece		swap		
Lytras et al(b) [74]	• Cross-sectional in Greece	• 394	• RT-PCR mass testing on nasopharyngeal swaps	• N/A	• Twenty-five (6.3%, 95% CI: 4.1–9.2%) positive asymptomatic cases
Lytras et al(c) [74]	• Cross-sectional in Greece	• 32	• RT-PCR mass testing on nasopharyngeal swap	• N/A	• Two (6.3%, 95% CI: 0.8–20.8%) positive asymptomatic cases
Hoehl et al [75]	• Cross-sectional in Germany	• 114	• RT-PCR mass testing on nasopharyngeal swap and sputum	• N/A	• Two (1.8%) of 114 asymptomatic passengers tested positive. All 11 symptomatic patients tested negative. • Symptom-based testing failed to detect SARS-CoV-2 patients.
Cao et al [76]	• Cross-sectional in China	• 9,899,828	• Citywide mass testing using TR-PCR on nasopharyngeal and throat swabs	• N/A	• No symptomatic case was found compared to 300 asymptomatic cases (0.303/10,000, 95% CI; 0.270–0.339/10,000)
Baggett et al [77]	• Cross-sectional in the USA	• 408	• Mass testing • Symptom screening (on nasopharyngeal swaps)	• N/A	• 147 (36.0%) subjects tested positive, of whom 87.8% were asymptomatic
Imbert et al	• Cross-section	• 210	• Mass RT-PCR testing on	• N/A	• Fifty-two (52%) of tested residents were

Study	Design/ setting	Sample	Strategies	Assumptions	Main findings
[78]	al in the USA		nasopharyngeal specimens		asymptomatic. This occurred when registered incidence was 5.1 case per 100,000

[a]Mass testing of individuals with high contact rates (at-risk group)

[b]Reverse transcription-polymerase chain reaction
