# Supplementary material for: Mass Testing With Contact Tracing Compared to Test and Trace for the Effective Suppression of COVID-19 in the United Kingdom: Systematic Review
Source: JMIRx Med. 2021 Apr 12;2(2):e27254. doi: 10.2196/27254 (PMC8045129; doi:10.2196/27254)
Supplement: Multimedia Appendix 2 [file xmed_v2i2e27254_app2.pdf]

Table S1: Characteristics of Excluded Studies

Study	Design/ Outcome	Sample/ setting	Strategies	Reason for exclusion
Maslov [79]	• Logical description<br>• Economic benefits	• Non	• Random mass testing	• No comparator
Peto et al [80]	• Modeling study<br>• Reproduction number<br>• Number of daily tests	• Hypothetical sample in the<br>• UK	• Weekly mass test and trace using isothermal single-step reverse transcription-polymerase chain reaction (RT-PCR)	• No comparator
Domenico et al [81]	• Modeling study<br>• Lockdown impact<br>• Number of contacts	• Age profile data of Ile-de- France and<br>• 2012 social contact matrix	• School closure<br>• Employee telework from home<br>• Senior isolation (high-risk group)<br>• Lockdown and non-essential activity ban<br>• Case isolation with large-scale testing	• Unsuitable comparator
Quilty et al [82]	• Modeling study<br>• Infected travelers	• Air travelers	• Symptoms screening	• Unsuitable design
Gostic et al [83]	• Modeling study<br>• Screening outcome and missed cases	• A hypothetical population of infected travelers	• Symptomatic but not aware of exposure risk<br>• Aware of exposure risk but without detectable symptoms<br>• Symptomatic and aware that exposure may have occurred<br>• Neither symptomatic nor aware of exposure risk	• Lack of intervention

Study	Design/ Outcome	Sample/ setting	Strategies	Reason for exclusion
Kucharski et al [84]	• Modeling study<br>• Reduction in transmission<br>• Daily contacts quarantined	• 40,162 participants and BBC 2017-18 social contact dataset in the UK	• No control measures.<br>• Self-isolation of symptomatic cases<br>• Household quarantine<br>• Quarantine of work or school contacts<br>• Manual tracing of acquaintances<br>• Manual tracing of all contacts<br>• App-based tracing<br>• Mass testing<br>• Daily limit of other setting contacts	• Unsuitable design. No comparison
Kirshblum et al [85]	• Retrospective study<br>• Test results and symptoms onset	• 103 admitted patients in the Rehabilitation hospital in the USA	• Analysis of samples collected at the time of admission	• Unsuitable design
Firth et al [86]	• Modeling study<br>• Number of tests<br>• Number of contacts	• 468 real-world social network data in the UK	• Outbreak progress under no intervention<br>• Outbreak progress under case isolation<br>• Outbreak progress under primary contact tracing<br>• Outbreak progress under secondary contact tracing	• Contact tracing limited to symptom-based testing
Keeling et al [87]	• Cross-sectional survey<br>• Tracing efficacy	• More than 5802 subjects reporting more	• N/A	• Contact tracing limited to

Study	Design/ Outcome	Sample/ setting	Strategies	Reason for exclusion
	• Distribution of secondary cases	than 50,000 contacts in the UK		symptom-based testing
Bilinski et al [88]	• Modeling study<br>• % reduction in reproduction number (R)	• Hypothetical in the US	• Symptom testing with 30% isolation and quarantine.<br>• Test all individuals, with 30% isolation and quarantine.<br>• Symptom testing with 60% isolation and quarantine.<br>• Test all individuals, with 60% isolation and quarantine.<br>• Symptom testing, with 90% isolation and quarantine<br>• Test all individuals, with 90% isolation and quarantine	• Contact tracing limited to symptom-based testing
Kretzschmar et al [89]	• Modeling study<br>• Reduction in the reproduction number	• Hypothetical sample in the<br>• Netherlands	• Conventional contact tracing<br>• Mobile app contact tracing<br>• Physical distancing strategy<br>• Testing and isolation of cases without tracing contacts	• Contact tracing limited to symptom-based testing
Skoll et al [90]	• Non-systematic review<br>• Role of technology, barriers, and scale-up strategies	• N/A	• Digital contact tracing and mass testing	• Unsuitable design
Kerr et al [91]	• Modeling study<br>• Feasibility of control strategies	• Demographic, mobility, and epidemiological	• Test and trace (testing, contact tracing, and quarantine)	• Limited to control

Study	Design/ Outcome	Sample/ setting	Strategies	Reason for exclusion
		data of Seattle in the USA		
Panovska-Griffiths et al [92]	• Modeling study<br>• Reduction in the reproduction number	• Modeled sample in the UK	• Full-time schooling<br>• Part-time weekly rota system of 50% each schooling<br>• 68% contact tracing with no scale-up in testing<br>• 68% contact tracing with sufficient testing<br>• 40% contact tracing with sufficient testing	• No suitable comparison
Hellewell et al [93]	• Modeling study<br>• Onward transmission	• Modeled sample in the UK	• 5, 20, and 40 initial cases of the outbreak<br>• 0, 0.2, 0.4, 0.6, 0.8 and 1 probabilities of tracing a contact<br>• Short symptom onset to isolation<br>• Long symptom onset to isolation	• Contact tracing limited to symptom-based testing
Ferretti et al [94]	• Modeling study<br>• Basic reproduction number (R)<br>• Generation time	• Pair of 40 hypothetical recipients in Singapore	• Symptomatic transmission<br>• Presymptomatic transmission<br>• Asymptomatic transmission<br>• Environmental transmission<br>• Isolating symptomatic persons<br>• Tracing the contacts of symptomatic cases and quarantining	• Contact tracing limited to symptom-based testing
Min et al [95]	• Modeling study<br>• Epidemic size	• Daily COVID-19 reported cases (Feb12-	• Social distancing among adults<br>• Spring semester postponement	• No suitable comparator

Study	Design/ Outcome	Sample/ setting	Strategies	Reason for exclusion
	• Effective contact rate	March3) in Korea	• Intensive contact tracing<br>• Large-scale diagnostic testing	
He et al [96]	• Modeling study<br>• Required resources<br>• Effect on R	• 40,162 BBC pandemic data in the UK	• Symptom-based contact tracing<br>• Test-based contact tracing<br>• Testing of asymptomatic contacts	• Contact tracing limited to symptom-based testing
Goscé et al [97]	• Modeling study	• PHE[a], NHS[b], and TfL[c] data<br>• Royal Borough of Kensington and Chelsea (RBKC) in the UK	• Isolation of RBKC residents from the rest of the city<br>• Removal of lockdown<br>• Weekly testing (business reopens but people work from home)<br>• Shielding 60+ age group with the lifting of lockdown<br>• Combined universal testing and use of face coverings with no lockdown.<br>• - Universal testing, contact tracing and isolation, lockdown	• Unsuitable design
Li et al [98]	• Descriptive study	• N/A	• Containment<br>• Suppression	• Unsuitable design
Kennedy-Shaffer et al [99]	• Modeling study<br>• Reduction in transmissions	• Unknown	• Hypothetical rapid test<br>• Transmission tracing<br>• Full isolation of all contacts of cases<br>• Isolate contacts with positive test results	• Unsuitable design
Campbell et al [100]	• Cross sectional	• 41,751	• Systematic trace and test contacts	• No suitable comparator

Study	Design/ Outcome	Sample/ setting	Strategies	Reason for exclusion
	• Cost, human resource and lab capacity	• COVID-19 contacts, the staff of hospitals, health centers, care homes & essential businesses, school children & staff in Canada	• Test all staff in acute care hospitals. • Test all community health workers and staff/residents of long-term care homes • Test all major public and interpersonal contact essential workers • Test all children and staff of schools	
Cleevely et al [101]	• Modeling study	• Hypothetical sample in the UK	• Stratified periodic sample testing • Universal random testing	• Unsuitable comparator
Yokota et al [102]	• Diagnostic tests • The utility of nucleic acid amplification	• 1924 asymptomatic persons in Japan	• Nasopharyngeal swap-based (NPS) RT-PCR test • Saliva-based PCR test	• Unsuitable design
Eilersen & Sneppen [103]	• Modeling study • Quarantine measures • Cost-effectiveness	• Hypothetical sample	• No intervention • Reduced work contacts by 75% • Reduced social contacts by 75% • Infection probability reduced by 50% • Workplace size reduced by half • Infection probability plus workplace size reduced	• Limited to control
Altawalah et al [104]	• Cross-sectional study • Detection of SARS-CoV-2 in saliva	• 891 suspects in Kuwait	• Nasopharyngeal swap-based (NPS) RT-PCR test • Saliva-based PCR test	• Unsuitable design

Study	Design/ Outcome	Sample/ setting	Strategies	Reason for exclusion
Dollard et al [105]	• Diagnostic test<br>• COVID-19 infections	• 298 air travelers in the USA	• Reverse transcription-polymerase chain reaction (RT-PCR)	• Asymptomatic proportions unknown
Telford et al [106]	• Cross-sectional<br>• Timing of mass testing	• 5671 residents & staff in 28 long term care facilities in the USA	• Mass RT-PCR test	• Asymptomatic proportion unknown
Bosetti et al [107]	• Modeling study<br>• Impact of intervention	• Real-time COVID-19 data in France	• Mass testing	• No comparator

[a] PHE = Public Health England
[b] NHS = National Health Service
[c] TfL = Transport for London
