# Supplementary material for: Mass Testing With Contact Tracing Compared to Test and Trace for the Effective Suppression of COVID-19 in the United Kingdom: Systematic Review
Source: JMIRx Med. 2021 Apr 12;2(2):e27254. doi: 10.2196/27254 (PMC8045129; doi:10.2196/27254)
Supplement: Multimedia Appendix 3 [file xmed_v2i2e27254_app3.pdf]

Table S1: Quality Assessment of Modeling Studies

Relevance and Credibility of Modeling Studies for Informing Health Care Decision Making															
Study	Relevance				Credibility										
					Validation			Design	Data	Analysis		Reporting	Interpretation	Conflict of interest	
	Is the population relevant?	Are any critical interventions missing?	Are any relevant outcomes missing?	Is the context (settings and circumstances) applicable?	Is external validation of the model sufficient to make its results credible for your decision?	Is internal verification of the model sufficient to make its results credible for your decision?	Does the model have sufficient face validity to make its results credible for your decision?	Is the design of the model adequate for your decision problem?	Are the data used in populating the model suitable for your decision problem?	Were the analysis performed using the model adequate to inform your decision problem?	Was there an adequate assessment of the effects of uncertainty?	Was the reporting of the model adequate to inform your decision problem?	Was the interpretation of the results fair and balanced?	Were there any potential conflicts of interest?	If there were potential conflicts of interest, were steps taken to address these?
**Effectiveness**															
Emery et al [44]	No	No	No	No	Yes	Yes	Yes	Yes	Yes	Yes	Yes	Yes	Yes	No	N/A
Grassly et al [45]	Yes	No	No	Yes	Yes	Yes	Yes	Yes	Yes	Yes	Yes	Yes	Yes	No	N/A

Relevance and Credibility of Modeling Studies for Informing Health Care Decision Making															
Study	Relevance				Credibility										
					Validation			Design	Data	Analysis		Reporting	Interpretation	Conflict of interest	
	Is the population relevant?	Are any critical interventions missing?	Are any relevant outcomes missing?	Is the context (settings and circumstances) applicable?	Is external validation of the model sufficient to make its results credible for your decision?	Is internal verification of the model sufficient to make its results credible for your decision?	Does the model have sufficient face validity to make its results credible for your decision?	Is the design of the model adequate for your decision problem?	Are the data used in populating the model suitable for your decision problem?	Were the analysis performed using the model adequate to inform your decision problem?	Was there an adequate assessment of the effects of uncertainty?	Was the reporting of the model adequate to inform your decision problem?	Was the interpretation of the results fair and balanced?	Were there any potential conflicts of interest?	If there were potential conflicts of interest, were steps taken to address these?
Tsou et al [46]	No	No	No	No	Yes	No	Yes	Yes	No	No	Yes	Yes	Yes	Can't answer	Not reported
Mizumoto et al [47]	No	Yes	No	No	Yes	No	Yes	Yes	Yes	Yes	No	Yes	Yes	No	N/A
Sasmita et al [48]	No	No	No	No	Yes	No	Yes	Yes	No	No	Yes	Insufficient infor	Yes	No	N/A

Relevance and Credibility of Modeling Studies for Informing Health Care Decision Making															
**Study**	**Relevance**				**Credibility**										
					**Validation**			**Design**	**Data**	**Analysis**		**Reporting**	**Interpretation**	**Conflict of interest**	
	**Is the population relevant?**	**Are any critical interventions missing?**	**Are any relevant outcomes missing?**	**Is the context (settings and circumstances) applicable?**	**Is external validation of the model sufficient to make its results credible for your decision?**	**Is internal verification of the model sufficient to make its results credible for your decision?**	**Does the model have sufficient face validity to make its results credible for your decision?**	**Is the design of the model adequate for your decision problem?**	**Are the data used in populating the model suitable for your decision problem?**	**Were the analysis performed using the model adequate to inform your decision problem?**	**Was there an adequate assessment of the effects of uncertainty?**	**Was the reporting of the model adequate to inform your decision problem?**	**Was the interpretation of the results fair and balanced?**	**Were there any potential conflicts of interest?**	**If there were potential conflicts of interest, were steps taken to address these?**
												matio n			
Moghadas et al [49]	No	No	No	No	No	No	Yes	Yes	No	Yes	Yes	Yes	Yes	No	N/A
Bracis et al [50]	No	No	No	No	Yes	Yes	Yes	Yes	Yes	Yes	Yes	Yes	Yes	No	N/A

Relevance and Credibility of Modeling Studies for Informing Health Care Decision Making														
Study	Relevance				Credibility									
					Validation			Design	Data	Analysis		Reporting	Interpretation	Conflict of interest
	Is the population relevant?	Are any critical interventions missing?	Are any relevant outcomes missing?	Is the context (settings and circumstances) applicable?	Is external validation of the model sufficient to make its results credible for your decision?	Is internal verification of the model sufficient to make its results credible for your decision?	Does the model have sufficient face validity to make its results credible for your decision?	Is the design of the model adequate for your decision problem?	Are the data used in populating the model suitable for your decision problem?	Were the analysis performed using the model adequate to inform your decision problem?	Was there an adequate assessment of the effects of uncertainty?	Was the reporting of the model adequate to inform your decision problem?	Was the interpretation of the results fair and balanced?	Were there any potential conflicts of interest?	If there were potential conflicts of interest, were steps taken to address these?
Pollmann et al [51]	Yes	No	No	Yes	No	No	Yes	Yes	Not enough information	Yes	Yes	Yes	Yes	Can't answer	Not reported
Hill et al [52]	Yes	No	No	Yes	Yes	Yes	Yes	Yes	Yes	Yes	Yes	Yes	Yes	No	N/A
Gorji et al [53]	No	No	No	No	No	Yes	Yes	Yes	No	Yes	Yes	Yes	Yes	Can't	Not reported

Relevance and Credibility of Modeling Studies for Informing Health Care Decision Making															
Study	Relevance				Credibility										
					Validation			Design	Data	Analysis		Reporting	Interpretation	Conflict of interest	
	Is the population relevant?	Are any critical interventions missing?	Are any relevant outcomes missing?	Is the context (settings and circumstances) applicable?	Is external validation of the model sufficient to make its results credible for your decision?	Is internal verification of the model sufficient to make its results credible for your decision?	Does the model have sufficient face validity to make its results credible for your decision?	Is the design of the model adequate for your decision problem?	Are the data used in populating the model suitable for your decision problem?	Were the analysis performed using the model adequate to inform your decision problem?	Was there an adequate assessment of the effects of uncertainty?	Was the reporting of the model adequate to inform your decision problem?	Was the interpretation of the results fair and balanced?	Were there any potential conflicts of interest?	If there were potential conflicts of interest, were steps taken to address these?
														answer	
Alsing et al [54]	Yes	No	No	Yes	Yes	Not enough information	Yes	Yes	No	Yes	Yes	Yes	Yes	Can't answer	Not reported
**Cost-Effectiveness**															

Relevance and Credibility of Modeling Studies for Informing Health Care Decision Making															
Study	Relevance				Credibility										
					Validation			Design	Data	Analysis		Reporting	Interpretation	Conflict of interest	
	Is the population relevant?	Are any critical interventions missing?	Are any relevant outcomes missing?	Is the context (settings and circumstances) applicable?	Is external validation of the model sufficient to make its results credible for your decision?	Is internal verification of the model sufficient to make its results credible for your decision?	Does the model have sufficient face validity to make its results credible for your decision?	Is the design of the model adequate for your decision problem?	Are the data used in populating the model suitable for your decision problem?	Were the analysis performed using the model adequate to inform your decision problem?	Was there an adequate assessment of the effects of uncertainty?	Was the reporting of the model adequate to inform your decision problem?	Was the interpretation of the results fair and balanced?	Were there any potential conflicts of interest?	If there were potential conflicts of interest, were steps taken to address these?
Paltiel et al [56]	No	No	No	No	No	No	Yes	Yes	No	Yes	Yes	Yes	Yes	No	N/A
