# Supplementary material for: Mass Testing With Contact Tracing Compared to Test and Trace for the Effective Suppression of COVID-19 in the United Kingdom: Systematic Review
Source: JMIRx Med. 2021 Apr 12;2(2):e27254. doi: 10.2196/27254 (PMC8045129; doi:10.2196/27254)
Supplement: Multimedia Appendix 4 [file xmed_v2i2e27254_app4.pdf]

Table S1: Quality Assessment of Cohort Study

Critical Appraisal Skills Program (CASP) Tool														
	Are the results of the study valid?								What are the results?					
Study	Did the study address a clearly focused issue?	Was the cohort recruited in an acceptable way?	Was the exposure accurately measured to minimise bias?	Was the outcome accurately measured to minimise bias?	Have the authors identified all important confounding factors?	Have they taken account of the confounding factors in the design and/or analysis?	Was the follow up of subjects complete enough?	Was the follow up of subjects long enough?	What are the results of this study[a]?	How precise are the results?	Do you believe the results?	Can the results be applied to the local population?	Do the results of this study fit with other available evidence?	What are the implications of this study for practice?
**Effectiveness**														
Porru et al [57]	Yes	Yes	Yes	Yes[b]	No[c]	Yes	Yes[d]	Yes	A total of 238 workers had a positive test with a cumulative incidence of 4.0%. A third of positive cases were symptomatic while 2.3% were asymptomatic.	Cumulative incidence was 4.0% (95% CI: 3.5–4.5%). The risk odds ratio in medical wards was 2.7 (95% CI: 1.9–	Yes	No[e]	Yes	Yes[f]

Critical Appraisal Skills Program (CASP) Tool														
	Are the results of the study valid?								What are the results?					
Study	Did the study address a clearly focused issue?	Was the cohort recruited in an acceptable way?	Was the exposure accurately measured to minimise bias?	Was the outcome accurately measured to minimise bias?	Have the authors identified all important confounding factors?	Have they taken account of the confounding factors in the design and/or analysis?	Was the follow up of subjects complete enough?	Was the follow up of subjects long enough?	What are the results of this study[a]?	How precise are the results?	Do you believe the results?	Can the results be applied to the local population?	Do the results of this study fit with other available evidence?	What are the implications of this study for practice?
									Incidence among the exposed was 40.5% and 0.5% among the non-exposed group	3.9) and 4.3 (95% CI: 2.4–7.6) in health services				

[a] The absolute risk reduction was unreported

[b] Neither subjects nor outcome assessors were blinded.

[c] Ethnicity of healthcare workers might have been important.

[d] No report on whether there was any loss to follow-up or not.

[e] Limited applicability due to study population and contextual differences

[f] Study highlights the importance of the intervention in a healthcare setting which can apply to any context.
