# Supplementary material for: Mass Testing With Contact Tracing Compared to Test and Trace for the Effective Suppression of COVID-19 in the United Kingdom: Systematic Review
Source: JMIRx Med. 2021 Apr 12;2(2):e27254. doi: 10.2196/27254 (PMC8045129; doi:10.2196/27254)
Supplement: Multimedia Appendix 5 [file xmed_v2i2e27254_app5.pdf]

Table S1: Quality Assessment of Cross-sectional Studies

Specialist Unit for Review Evidence (SURE) Profile Table												
Study	Is the study design clearly stated?	Does the study address a clearly focused question?	Are the setting, locations and relevant dates provided?	Were participants fairly selected?	Are participant characteristics provided?	Are the measures of exposures & outcomes appropriate?	Is there a description of how the study size was arrived at?	Are the statistical methods well described?	Is information provided on participant eligibility?	Are the results well described?	Is any sponsorship/conflict of interest reported?	Did the authors identify any limitations and, if so, are they captured above?
**Effectiveness**												
Hagan et al [55]	No	Yes	No	Can't tell	Yes	No	No	No	No	Yes	Yes	Yes
**Asymptomatic proportion**												
Nishiura et al [58]	No	Yes	Yes	Yes	No	No	N/A	No	N/A	No	Yes	No
Treibel et al [59]	No	Yes	Yes	Can't tell	No	No	No	No	No	Yes	Yes	No
Brown et al [61]	Yes	Yes	Yes	Yes	Yes	Yes	No	Yes	Yes	Yes	Yes	Yes

Specialist Unit for Review Evidence (SURE) Profile Table												
Study	Is the study design clearly stated?	Does the study address a clearly focused question?	Are the setting, locations and relevant dates provided?	Were participants fairly selected?	Are participant characteristics provided?	Are the measures of exposures & outcomes appropriate?	Is there a description of how the study size was arrived at?	Are the statistical methods well described?	Is information provided on participant eligibility?	Are the results well described?	Is any sponsorship/conflict of interest reported?	Did the authors identify any limitations and, if so, are they captured above?
Graham et al [62]	Yes	Yes	Yes	Can’t tell	Yes	Yes	Yes	Yes	No	Yes	Yes	Yes
Abeysuriya et al [60]	Yes	Yes	Yes	Yes	Yes	No	N/A	Yes	N/A	Yes	Yes	Yes
Arons et al [63]	Yes	Yes	Yes	Can’t tell	Yes	Yes	No	No	No	Yes	No	Yes
Jameson et al [64]	No	Yes	Yes	Yes	No	No	N/A	No	No	No	Yes	No
Callaghan et al [65]	Yes	Yes	Yes	Can’t tell	Yes	Yes	No	No	No	Yes	Yes	Yes
Louie et al [66]	Yes	Yes	Yes	Can’t tell	Yes	Yes	No	No	No	Yes	Yes	Yes
Gudbjartsson et al [67]	Yes	Yes	Yes	Can’t tell	Yes	Yes	No	Yes	No	Yes	Yes	No
Reid et al [68]	Yes	Yes	Yes	Yes	Yes	No	N/A	Yes	N/A	No	Yes	No

Specialist Unit for Review Evidence (SURE) Profile Table

Study	Is the study design clearly stated?	Does the study address a clearly focused question?	Are the setting, locations and relevant dates provided?	Were participants fairly selected?	Are participant characteristics provided?	Are the measures of exposures & outcomes appropriate?	Is there a description of how the study size was arrived at?	Are the statistical methods well described?	Is information provided on participant eligibility?	Are the results well described?	Is any sponsorship/conflict of interest reported?	Did the authors identify any limitations and, if so, are they captured above?
Lavezzo et al [69]	Yes	Yes	Yes	Yes	No	Yes	Yes	Yes	Yes	Yes	Yes	Yes
Kimball et al [70]	No	Yes	Yes	Can’t tell	Yes	Yes	No	No	No	No	Yes	Yes
Olalla et al [71]	Yes	Yes	Yes	Can’t tell	Yes	Yes	No	Yes	No	Yes	Yes	Yes
Guery et al [72]	Yes	Yes	Yes	Yes	Yes	Yes	N/A	No	N/A	No	Yes	Yes
Roxby et al [73]	No	Yes	Yes	Can’t tell	Yes	Yes	No	No	No	No	Yes	Yes
Lytras et al [74]	No	Yes	Yes	Can’t tell	Yes	No	No	No	No	No	Yes	No
Hoehl et al [75]	No	Yes	Yes	Can’t tell	No	No	No	No	No	No	No	No
Cao et al [76]	Yes	Yes	Yes	Yes	Yes	Yes	Yes	Yes	Yes	Yes	Yes	Yes

Specialist Unit for Review Evidence (SURE) Profile Table												
Study	Is the study design clearly stated?	Does the study address a clearly focused question?	Are the setting, locations and relevant dates provided?	Were participants fairly selected?	Are participant characteristics provided?	Are the measures of exposures & outcomes appropriate?	Is there a description of how the study size was arrived at?	Are the statistical methods well described?	Is information provided on participant eligibility?	Are the results well described?	Is any sponsorship/conflict of interest reported?	Did the authors identify any limitations and, if so, are they captured above?
Baggett et al [77]	No	Yes	Yes	Can't tell	Yes	Yes	No	No	No	Yes	Yes	Yes
Imbert et al [78]	No	Yes	Yes	Can't tell	Yes	No	No	Yes	No	No	Yes	Yes
