# Supplementary material for: Mass Testing With Contact Tracing Compared to Test and Trace for the Effective Suppression of COVID-19 in the United Kingdom: Systematic Review
Source: JMIRx Med. 2021 Apr 12;2(2):e27254. doi: 10.2196/27254 (PMC8045129; doi:10.2196/27254)
Supplement: Multimedia Appendix 6 [file xmed_v2i2e27254_app6.pdf]

Table S1: Certainty of Evidence for the Primary Objective

GRADE Evidence Profile: Mass Testing and Contact Tracing compared to Conventional Test and Trace								
	Quality of Evidence Factors					Direction of Effect Summary of Findings<br>Conventional Test and Trace / Mass Test and Trace<br>Control of SARS-CoV-2/COVID-19 Transmissions		Quality of evidence
No of Studies (Design)	Study bias	Heterogeneity	Indirectness	Imprecision	Publication Bias			
**Effectiveness**								
n=11 (Modeling studies)	Serious[a]	Serious[b]	Serious[c]	Serious[d]	Unlikely	**Emery et al** [44]<br>53% (95% Posterior Interval, PI: 51-56%) of asymptomatic carriers under symptom-based testing went undetected compared to mass testing.	↑	●○○○
						**Grassly et al** [45]<br>Test and trace will reduce R[e] by 8% (95% Uncertainty Interval 5–11) for 50% coverage and 48-hour sample-quarantine delay, compared to mass PCR testing	↓	
						Tsou et al [46]<br>Symptom-based testing prevented no subclinical case while symptom-based plus at-risk group testing prevented 40%, 60%, and 80% of subclinical cases	↑	
						**Mizumoto et al** [47]<br>A total of 634 detected due to mass testing compared to 306 symptomatic	↑	

GRADE Evidence Profile: Mass Testing and Contact Tracing compared to Conventional Test and Trace								
	Quality of Evidence Factors					Direction of Effect Summary of Findings		
No of Studies (Design)	Study bias	Heterogeneity	Indirectness	Imprecision	Publication Bias	Conventional Test and Trace / Mass Test and Trace<br>Control of SARS-CoV-2/COVID-19 Transmissions		Quality of evidence
						cases that would have been detected through the symptom-based approach		
						**Sasmita et al** [48]<br>Contact tracing (test trace) combined with other measures showed to be more effective than mass testing combined with other measures in outbreak prediction	↓	
						**Moghadas et al** [49]<br>Symptom-based test and trace must be combined with testing irrespective of symptomology	↑	
						**Bracis et al** [50]<br>Symptom test and trace was more effective than mass testing in reducing daily deaths and when aiming for 70% post-COVID-19 physical interactions	↓	
						**Pollmann et al** [51]<br>Mass random testing and contact tracing can control the outbreak as oppose to contact tracing (test and trace)	↑	
						**Hill et al** [52]	↑	

GRADE Evidence Profile: Mass Testing and Contact Tracing compared to Conventional Test and Trace								
No of Studies (Design)	Quality of Evidence Factors					Direction of Effect Summary of Findings		Quality of evidence
	Study bias	Heterogeneity	Indirectness	Imprecision	Publication Bias	Conventional Test and Trace / Mass Test and Trace		
						Control of SARS-CoV-2/COVID-19 Transmissions		
						Regular mass testing and contact tracing reduced infections by more than 50% compared to when there is no mass testing		
						**Gorji et al** [53] Mass testing (about 166 per 100,000) based on contact counting is more effective, reducing reproduction number from R = 2.4 to R = 1	↑	
						**Alsing at al** [54] Mass testing and contact tracing can contain 74% of the outbreak and get R below 1 more than contact tracing	↑	
**Effectiveness**								
n=1 (Cross-sectional)	Serious[f]	Unlikely	Serious[g]	Serious[h]	Unlikely	**Hagan et al** [55] Mass testing identified 8,239 (Range; 10-2193, Median=403) compared to 642 (Range: 2-181, Median=19) during symptom-based testing	↑	●○○○
**Cost-effectiveness**								
n=1	Serious[i]	Unlikely	Serious[j]	Serious[k]	Unlikely	**Paltiel et al** [56]	↑	●○○○

GRADE Evidence Profile: Mass Testing and Contact Tracing compared to Conventional Test and Trace								
	Quality of Evidence Factors					Direction of Effect Summary of Findings		
No of Studies (Design)	Study bias	Heterogeneity	Indirectness	Imprecision	Publication Bias	Conventional Test and Trace	Mass Test and Trace	Quality of evidence
						Control of SARS-CoV-2/COVID-19 Transmissions		
(Modeling study)						Mass testing/screening (every 1, 2, or 7days) was found to be more effective for R=3.5, 2.5, or 1.5 respectively, compared to symptom-based screening		

Favorable ↑ unfavorable ↓ Null effect ↔

[a] Internal validation for most studies and treatment of parameter/structural uncertainties unclear for some studies.

[b] Differences in study populations and settings. Lack of confidence intervals and statistical significance

[c] Population and settings in 5 studies were not representative

[d] No precise effect estimates in reported prediction in 8 studies.

[e] R = Reproduction number

[f] Possible methodological issues around subjects' recruitment and outcome measurements

[g] Unrepresentative population and setting

[h] Unreported effect estimates.

[i] No use of real-world data set and lack of clear external and internal validation process

[j] Unsuitable population and setting

[k] No precision in effect estimates.
