# Supplementary material for: Mass Testing With Contact Tracing Compared to Test and Trace for the Effective Suppression of COVID-19 in the United Kingdom: Systematic Review
Source: JMIRx Med. 2021 Apr 12;2(2):e27254. doi: 10.2196/27254 (PMC8045129; doi:10.2196/27254)
Supplement: Multimedia Appendix 7 [file xmed_v2i2e27254_app7.pdf]

Table S1: Details of Mass Testing

	Study	Test Details	Cases	Asym[a]	Sample	% in Cases	(95% CI)	% in Sample	(95% CI)
			2661	1084	9,942,878	40.7	(38.9–42.6)	0.0	(0.0–0.0)
**Asymptomatic proportion**									
	Porru et al [57]	Real-time PCR (Seegene AllplexTM2019-nCoV Assay) on oropharyngeal and nasopharyngeal swabs	238	109	5942	45.8	(39.3–52.4)	1.8	(1.5–2.2)
	Nishiura et al [58]	Reverse transcription polymerase chain reaction	13	4	565	30.8	(7.7–53.8)	0.7	(0.2–1.8)
	Treibel et al (staffw1) [59]		28	28	396	100	(87.6–100)	7.1	(4·9–10·0)
	Treibel et al (staffw2) [59]		14	14	284	100	(76.8–100)	4.9	(3·0–8·1)
	Treibel et al (staffw3) [59]		4	4	263	100	(39.8–100)	1.5	(0·6–3·8)
	Treibel et al (staffw4) [59]		4	4	267	100	(39 –100)	1.5	(0·6–3·8)
	Treibel et al (staffw5) [59]		3	3	269	100	(29.2–100)	1.1	(0·4–3 ·2)
	Abeysuriya et al [60]		7	6	180	85.7	(42.1–99.6)	3.3	(1.2–7.1)

	Study	Test Details	Cases	Asym[a]	Sample	% in Cases	(95% CI)	% in Sample	(95% CI)
			**2661**	**1084**	**9,942,878**	**40.7**	**(38.9–42.6)**	**0.0**	**(0.0–0.0)**
	Brown et al [61]	Applied Biosystems 7500 FAST system and ELITe InGenius (OSANG Healtcare)	23	4	1152	17.4	(5.0–38.8)	0.3	(0.1–0.9)
	Graham et al (residts) [62]	AusDiagnostics, Roche Cobas and Abbott RealTime SARS- CoV-2 assays	126	54	313	42.9	(34–52)	17.3	(13.2–21.9)
	Graham et al (staff) [62]		3	3	70	100	(29.2–100)	4.3	(1–9)
	Arons et al [63]	SARS-CoV-2 CDC assay protocol	48	27	76	56.3	(41.2–70.5)	35.5	(24.9–47.3)
	Jameson et al [64]	GeneXpert RT-PCR assay (Cepheid, Sunnyvale, CA)	0	0	121	-	-	0.0	(0.0–2.5)
	Callaghan et al [65]	RT-PCR[b] Diagnostic Panel	0	0	217	-	-	0.0	(0.0–1.2)
	Louie et al (redts-staff) [66]	Abbott m2000 RT-PCR	86	86	303	100	(95.8–100)	28.4	(23.4–33.8)
	Gudbjartssonet al (target1) [67]	TaqMan™ Fast Virus 1- step Master Mix, 2019-nCoV Assay kits v1 (Thermo Fisher)	177	24	1924	13.6	(8.9–19.5)	1.2	(0.8–1.9)
	Gudbjartsson et al(pop) [67]		87	51	10,797	58.6	(47.6–69.1)	0.5	(0.4–0.6)
	Gudbjartssonet al (target2) [67]		1044	59	7275	5.7	(4.3–7.2)	0.8	(0.6–1.0)
	Gudbjartssonet al(random) [67]		13	7	2283	53.8	(25.1–80.8)	0.3	(0.1–0.6)
	Reid et al [68]		5	5	2751	100	(47.8–100)	0.2	(0.1–0.2)

	Study	Test Details	Cases	Asym[a]	Sample	% in Cases	(95% CI)	% in Sample	(95% CI)
			2661	1084	9,942,878	40.7	(38.9–42.6)	0.0	(0.0–0.0)
	Lavezzo et al (surv1) [69]	TaqMan Fast Virus 1-Step Master Mix (Thermo Fisher)	73	29	2812	39.7	(28.5–51.9)	1.0	(0.7–1.5)
	Lavezzo et al (surv2) [69]		29	13	2343	44.8	(26.5–64.3)	0.6	(0.3–0.9)
	Kimball et al [70]	SARS-CoV-2 CDC assay protocol	23	13	76	56.5	(34.5–76.8)	17.1	(9.4–27.5)
	Olalla et al [71]	VIASURE SARS-CoV-2 from CerTest Biotec and LightMix Modular SARS-CoV (COVID19), Roche	2	1	498	50.0	(1.3–98.7)	0.2	(0–1.1)
	Guery et al [72]	Detection kit for 2019 novel coronavirus RNA, PCR Fluorescence Probing, (Daan Gene Co.)	3	2	136	66.7	(9.4–99.2)	1.5	(0.2–5.2)
	Roxby et al (redts-staffd1) [73]		5	3	142	60.0	(14.7–94.7)	2.1	(0.4–6.0)
	Roxby et al (redts-staff2) [73]		3	3	80	100	(29.2–100)	3.7	(0.8–10.6)
	Lytras et al (UK) [74]		13	13	357	100	(75.3–100)	3.6	(2.0–6.1)
	Lytras et al (Spain) [74]		25	25	394	100	(86.3–100)	6.3	(4.1–9.2)

	Study	Test Details	Cases	Asym[a]	Sample	% in Cases	(95% CI)	% in Sample	(95% CI)
			**2661**	**1084**	**9,942,878**	**40.7**	**(38.9–42.6)**	**0.0**	**(0.0–0.0)**
	Lytras et al (Turkey) [74]		2	2	32	100	(15.8–100)	6.3	(0.8–20.8)
	Hoehl et al [75]	LightMix Modular SARS and Wuhan CoV E-gene, and LightMix Modular Wuhan CoV RdRP-gen (TIB MOLBIOL)	2	2	114	100	(15.8–100)	1.8	(0.2–6.2)
	Cao et al [76]	Real-time fluorescence RT-PCR	300	300	9,899,828	100	(98.8–100)	0.0	0
	Baggett et al [77]		147	129	408	87.8	(81.3–92.6)	31.6	(27.1–36.4)
	Imbert et al [78]	Abbott m2000 RT-PCR	111	57	210	51.4	(41.7–61)	27.1	(21.3–33.1)

[a] Asym = Asymptomatic

[b] RT-PCR = Reverse transcription-polymerase chain reaction
